# Assessing DNA Barcodes for Species Identification in North American Reptiles and Amphibians in Natural History Collections

**DOI:** 10.1371/journal.pone.0154363

**Published:** 2016-04-26

**Authors:** E. Anne Chambers, Paul D. N. Hebert

**Affiliations:** 1 Department of Integrative Biology, University of Texas, Austin, Texas, United States of America; 2 Centre for Biodiversity Genomics, Biodiversity Institute of Ontario, University of Guelph, Guelph, Ontario, Canada; National Center for Biotechnology Information, UNITED STATES

## Abstract

**Background:**

High rates of species discovery and loss have led to the urgent need for more rapid assessment of species diversity in the herpetofauna. DNA barcoding allows for the preliminary identification of species based on sequence divergence. Prior DNA barcoding work on reptiles and amphibians has revealed higher biodiversity counts than previously estimated due to cases of cryptic and undiscovered species. Past studies have provided DNA barcodes for just 14% of the North American herpetofauna, revealing the need for expanded coverage.

**Methodology/Principal Findings:**

This study extends the DNA barcode reference library for North American herpetofauna, assesses the utility of this approach in aiding species delimitation, and examines the correspondence between current species boundaries and sequence clusters designated by the BIN system. Sequences were obtained from 730 specimens, representing 274 species (43%) from the North American herpetofauna. Mean intraspecific divergences were 1% and 3%, while average congeneric sequence divergences were 16% and 14% in amphibians and reptiles, respectively. BIN assignments corresponded with current species boundaries in 79% of amphibians, 100% of turtles, and 60% of squamates. Deep divergences (>2%) were noted in 35% of squamate and 16% of amphibian species, and low divergences (<2%) occurred in 12% of reptiles and 23% of amphibians, patterns reflected in BIN assignments. Sequence recovery declined with specimen age, and variation in recovery success was noted among collections. Within collections, barcodes effectively flagged seven mislabeled tissues, and barcode fragments were recovered from five formalin-fixed specimens.

**Conclusions/Significance:**

This study demonstrates that DNA barcodes can effectively flag errors in museum collections, while BIN splits and merges reveal taxa belonging to deeply diverged or hybridizing lineages. This study is the first effort to compile a reference library of DNA barcodes for herpetofauna on a continental scale.

## Introduction

Reptiles and amphibians are collectively the most threatened groups of vertebrates. Moreover, their species richness is currently underestimated as the rate of new and cryptic species discovery remains high [1–4]. Traditional morphology-based methods for species delimitation and description are time-consuming, and results are often unclear. In some cases, this provokes the inappropriate prioritization of species for conservation and in other cases, this slow work flow leads to the disappearance of a species before its description [5–7]. The rapid and objective documentation of diversity in the herpetofauna will facilitate species discovery, and will help to ensure that conservation programs are properly targeted. Therefore, there is an urgent need for a standardized protocol enabling rapid and effective species identification, especially given that conservation goals are based on species-level designations [8]. DNA barcoding, the preliminary identification of species using sequence diversity in a segment of the mitochondrial cytochrome *c* oxidase subunit 1 (COI) gene [9], has facilitated species delimitation and discovery in many organisms, including the herpetofauna [10–14]. However, no prior DNA barcoding study has aimed to acquire comprehensive coverage for the reptile and amphibian fauna of an entire continent. The Barcode Index Number (BIN) system [15] employs an algorithmic approach to objectively delineate sequence clusters that often correspond to species boundaries based on sequence variation at COI. Although the BIN system can aid species delimitation [16] and has been an effective tool for cryptic species discovery in various taxa [17,18], its performance remains to be thoroughly tested on vertebrates.

Canada and the United States (hereafter North America) host 292 amphibian and 348 reptilian species, approximately 8% of the global fauna [19]. North American taxa present an ideal opportunity to test the effectiveness of DNA barcoding because they have been the subject of intensive phylogenetic and morphological studies. Additionally, large collections of specimens are available, allowing for the sampling of rare or endangered species [20,21]. Because most reptilian and amphibian type specimens are more than a century old and many have been stored in formalin, the recovery of DNA sequences from them is problematic [22]. However, little effort has been made to recover DNA from the barcode region of formalin-fixed tissue through the recovery of partial fragments [23,24]. By coupling the analysis of tissue samples from museum specimens with the high-throughput workflows of DNA barcoding, this project performs a continent-wide analysis that integrates museum collections, morphological identifications, and DNA barcoding.

Reptiles and amphibians present an interesting challenge for species identification using DNA barcoding because introgressive hybridization and incomplete lineage sorting have resulted in barcode sharing by some closely related taxa [25–27]. Amphibians present an additional challenge as prior studies [26,28] have suggested that PCR amplification of COI is complicated by the presence of sequence variation at primer binding sites. Past attempts to test primers for the barcode region in amphibians have usually been limited to one genus or family, and have often examined taxa at a small geographic scale (e.g. [25–27,29,30]). Because COI has been adopted by the global research community as the barcode standard for the animal kingdom, a serious effort needs to be directed towards overcoming technical obstacles associated with barcoding herpetofauna. This project addresses this challenge by examining the efficacy of sequence recovery and species delimitation with DNA barcodes using the North American herpetofauna.

This study has the primary goal of compiling a reference library of DNA barcodes for the North American herpetofauna, as well as examining the correspondence between sequence clusters delineated by the BIN algorithm [15] and currently recognized species boundaries, the results of which provided an opportunity to detect labeling errors in museum collections. This study also tests the recovery of DNA sequence information from formalin-fixed specimens with primer sets that target short segments of the barcode region, and investigates tissue sample age and institution as factors that may influence sequence recovery from museum collections.

## Materials and Methods

### Specimen acquisition

Work began with the compilation of a species list for all reptiles and amphibians of North America using the resources provided by the Center for North American Herpetology (www.cnah.org). A total of 832 specimens from 814 species (576 reptiles, 169 species; 256 amphibians, 126 species) were subsequently obtained from frozen or ethanol-preserved tissue collections at seven museums, while 208 formalin-fixed samples (71 amphibian species, 52 reptilian species) were analyzed from the Smithsonian’s National Museum of Natural History and the Harvard Museum of Comparative Zoology. To examine the universality of the primer sets designed in this study, 55 specimens (32 species) of reptiles originating from outside North America were also examined. Two datasets provide specimen details, including photos, sequences and trace files; they can be retrieved on the Barcode of Life Data system (BOLD) using a DOI for North American herpetofauna (dx.doi.org/10.5883/DS-NAHERPS) and non-North American reptiles (dx.doi.org/10.5883/DS-EANAO) (www.boldsystems.org) [31], with sequence data also available on GenBank (S1 Table).

### DNA extraction, amplification, and sequencing

Tissue lysis, DNA extraction, PCR, and sequencing of all specimens followed standard protocols employed by the Canadian Centre for DNA Barcoding [32]. Dilution factors (S2 Table) and PCR regimes (S3 Table) were altered depending on the primer sets used. Because the AmphF2_t1+AmphR3_t1 primer set had the highest initial sequencing success overall and recovered full length barcodes, it was adopted for the initial round of PCR for all specimens (Table 1). In addition, the performance of existing primer sets for anurans and caudates [29] were tested on all amphibians. If the initial primer sets failed to generate an amplicon, two additional PCR reactions were performed which aimed to generate 307bp and 407bp amplicons (AmphF2_t1+MLepR2 and MLepF1+AmphR3_t1, respectively). Finally, when only one of these reactions generated a product, primer sets amplifying a 295bp or 189bp amplicon (MLepF2_t1+MicroLepR2 and AncientLepF2+MLepR2, respectively) were used with the goal of recovering a sequence that was sufficiently long (>487bp) to meet barcode compliance (Table 1). Due to the highly degraded state of DNA in formalin-fixed tissues, the reverse protocol was performed on these specimens, with primer sets generating the shortest amplicons (MLepF2_t1+MicroLepR2 and AncientLepF2+MLepR2) being run first. If successful, subsequent attempts at amplifying longer sequences were made using first AmphF2_t1+MLepR2 and MLepF1+AmphR3_t1, followed by AmphF2_t1+AmphR3_t1.

**Table 1:** Details for primer sets used in this study.

Primer sequence (5–3)	Name	Source
Forward		
TYT CWA CWA AYC AYA AAG AYA TCG G	Chmf4	[29]
AYT CAA CAA ATC ATA AAG ATA TTG G	COI-C02	[29]
T GTA AAA CGA CGG CCA GTT TCA ACW AAY CAY AAA GAY ATY GG	AmphF2_t1*	This study
GCT TTC CCA CGA ATA AAT AAT A	MLepF1	[73]
TGT AAA ACG ACG GCC AGT GCW TTC CCM CGW ATA AAT AAT ATA AG	MLepF2_t1*	[40]
ATT RRW RAT GAT CAA RTW TAT AAT	AncientLepF2	[40]
Reverse		
ACY TCR GGR TGR CCR AAR AAT CA	Chmr4	[29]
ACY TCR GGR TGA CCA AAA AAT CA	COI-C04	[29]
CA GGA AAC AGC TAT GAC TAD ACT TCW GGR TGD CCR AAR AAT CA	AmphR3_t1*	This study
GT TCA WCC WGT WCC WGC YCC ATT TTC	MLepR2	[40]
C AGG AAA CAG CTA TGA CGT AAT WGC WCC WGC TAR WAC WGG	MicroLepR2	[40]

*Use of M13 primers for sequencing reaction.

CodonCode Aligner version 3.7.1.2 (CodonCode Corporation) was used for Clustal W and manual sequence alignment. Sequences were translated to amino acids and examined for stop codons as a check for pseudogene amplification. Prior to uploading sequences acquired through the concatenation of several amplicons, each sequence was validated using the BOLD identification engine [31], as well as the Basic Local Alignment Search Tool (BLAST) at NCBI [33] to ensure that no chimeric sequences had been generated.

### Data analysis

Pairwise distances, inter- and intraspecific distance comparisons, sequence composition, and BINs were calculated using tools on BOLD [31], and further group-specific examinations were made using the SpeciesIdentifier package in the TaxonDNA program [34]. A neighbor-joining (NJ) tree [35] was constructed using pairwise sequence divergence estimated using the Kimura-2 parameter (K2P) distance model [36], and sequences were visually inspected for insertions and deletions using MEGA5 [37]. The relationship between maximum intraspecific sequence divergence and nearest neighbor divergence in conjunction with the NJ tree was then examined to detect potential errors in tissue samples analyzed from the Royal Ontario Museum.

Using linear regression, the collection dates for specimens (when available) were compared with sequence length to determine if sequence recovery decreased with age, with institution as an additional predictor variable [22,38]. Chi-square tests of homogeneity were used to determine whether there were differences in both overall sequencing success rates and barcode compliant sequence recovery between institutions for amphibians and reptiles separately. All linear regressions and chi-square tests of homogeneity were performed using R. DAMBE5 [39] was employed to compare the relative frequencies of transitions and transversions against K2P sequence divergences considering all three codon positions.

## Results

### Sequence recovery

A total of 730 sequences were recovered from 832 specimens (88% success) representing 274 species or 43% of the North American herpetofauna. Although most (533) sequences were barcode compliant (>487bp, <1%N), sequence lengths ranged from 123bp-658bp, reflecting the length of the target region for the different primer sets (S2 Table). Barcode compliant sequences were obtained from 38 of 55 specimens (69% success) of non-North American reptiles including representatives of 29 species using the AmphF2_t1+AmphR3_t1 primer set. The newly developed primer set (AmphF2_t1+AmphR3_t1) was the most successful for reptiles (S2 Table). However, the primer sets designed previously [29] performed best for amphibians, and successfully recovered barcodes from three specimens that were over 100 years old.

#### Formalin-fixed specimens

Five sequences from one reptile and four amphibians were recovered from the 208 formalin-fixed specimens. Although four of these sequences were not barcode compliant (sequence lengths ranged from 166bp-221bp) as they were amplified using the AnctLep+MLepR2 primer set [40], one barcode (*Rana hecksheri*, MCZ Herp A-37209) was amplified using the primers designed in this study (AmphF2_t1+AmphR3_t1). Not only was this sequence barcode compliant (length = 556bp), but it was assigned to a BIN providing a basis for the tentative identification of other specimens of this species.

### Factors influencing sequence recovery

**Fig 1:**
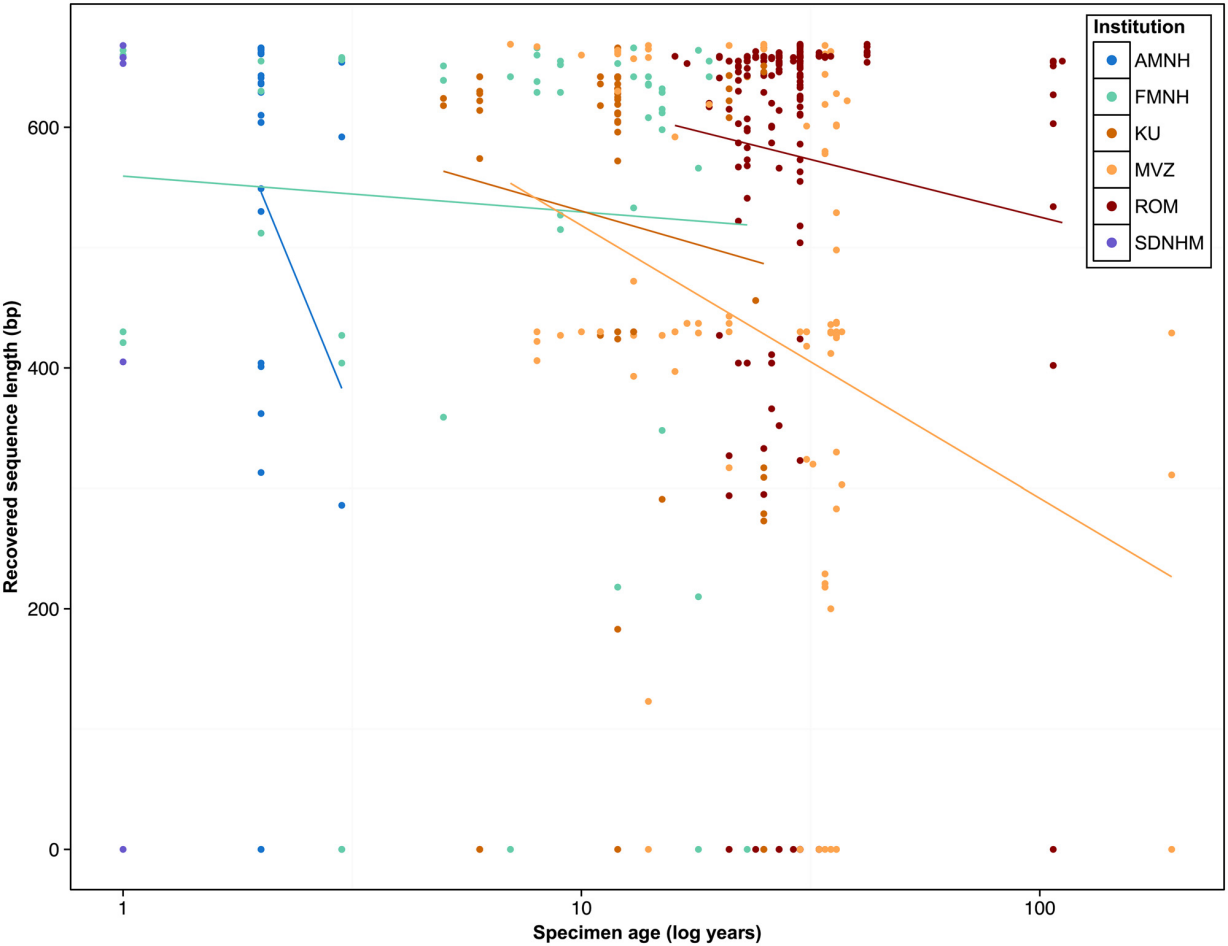
Relationship between sample age and recovered sequence length from six collections in reptiles and amphibians. AMNH: American Museum of Natural History; FMNH: Field Museum of Natural History; KU: University of Kansas Biodiversity Institute; MVZ: Museum of Vertebrate Zoology; ROM: Royal Ontario Museum; SDNHM: San Diego Natural History Museum; UAHC: University of Alabama, Alabama Museum of Natural History Herpetological Collection.

**Fig 2:**
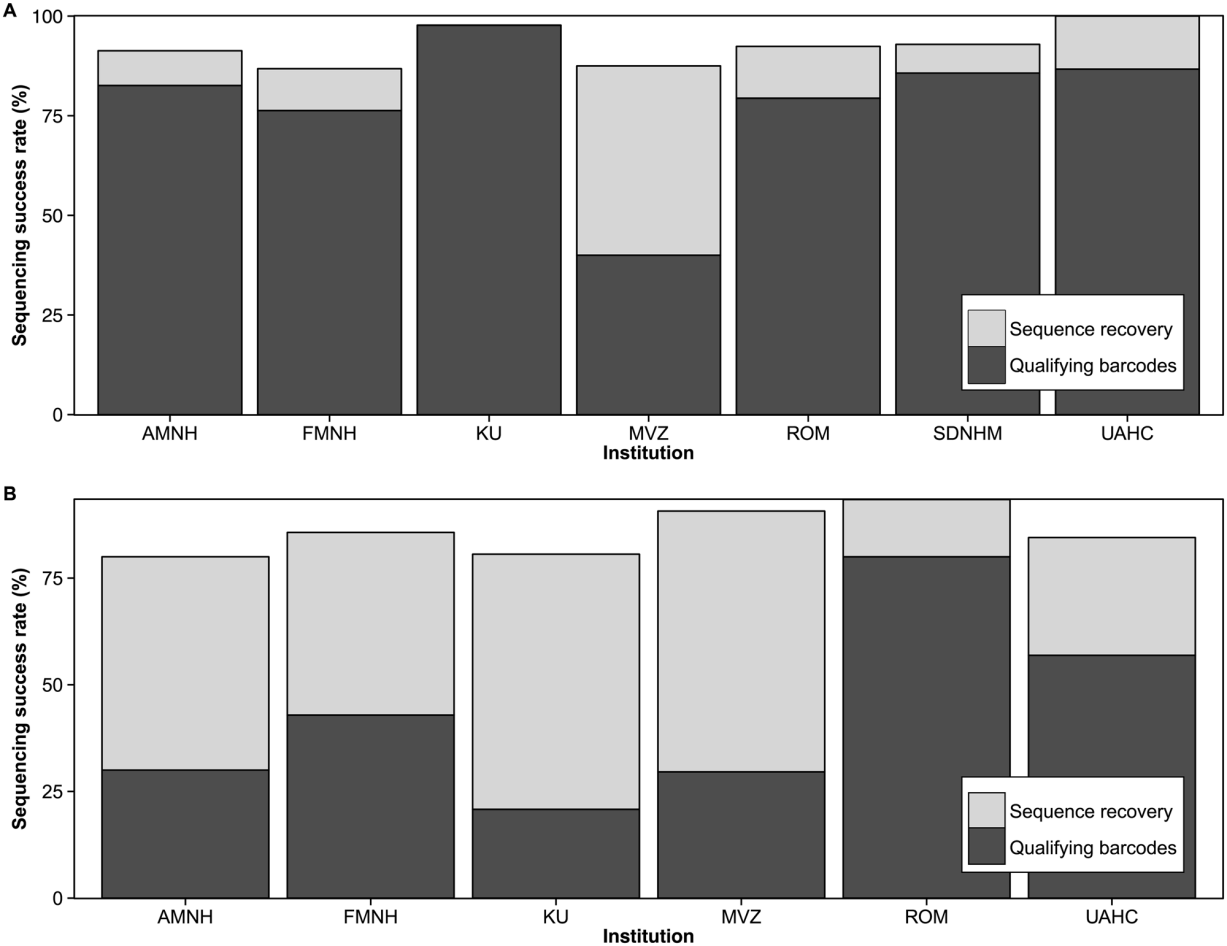
Sequence recovery in (A) amphibians and (B) reptiles from six collections. Qualifying barcodes are sequences which are ≥487bp.

A significant decrease in sequence length was observed with increasing specimen age (F statistic = 8.446 on 6 and 528 degrees of freedom, *p* <0.0001) (Fig 1). This relationship was unchanged even following the incorporation of institution as a predictor variable, although specimens from the Royal Ontario Museum had significantly higher sequence recovery than the other six institutions (*p* = 0.02311) (S4 Table). Chi-square tests of homogeneity revealed that although overall sequencing success was uniform across institutions in both classes (Amphibia: χ^2^ = 5.35, p = 0.37; Reptilia: χ^2^ = 7.02, *p* = 0.32), barcode compliant sequence recovery was significantly different among six institutions for amphibians (χ^2^ = 40.69, *p*<0.0001) and among all seven for reptiles (χ^2^ = 45.49, *p*<0.0001) (Fig 2). There was significantly lower success in overall sequence recovery (χ^2^ = 140.9, *p*<0.0001) as well as in recovery of barcode compliant sequences (χ^2^ = 108.5, *p*<0.0001) from amphibians than reptiles.

### COI sequence variation

Intra- and interspecific distances varied widely in reptiles and amphibians, with interspecific sequence divergence as low as 0% in both classes and intraspecific divergence as high as 21.22% in squamates. Barcode sharing was not observed between different species in either class. However, cases of low interspecific divergence (<2%) occurred in eight pairs of comparisons between two species of reptile (12%) and seven pairs of comparisons in amphibians (23%), with six of the latter cases belonging to the family Plethodontidae. Deep intraspecific divergence (>2%) was observed in 47 of 133 reptilian species (35%), but in only 10 of 62 amphibian species (16%).

**Fig 3:**
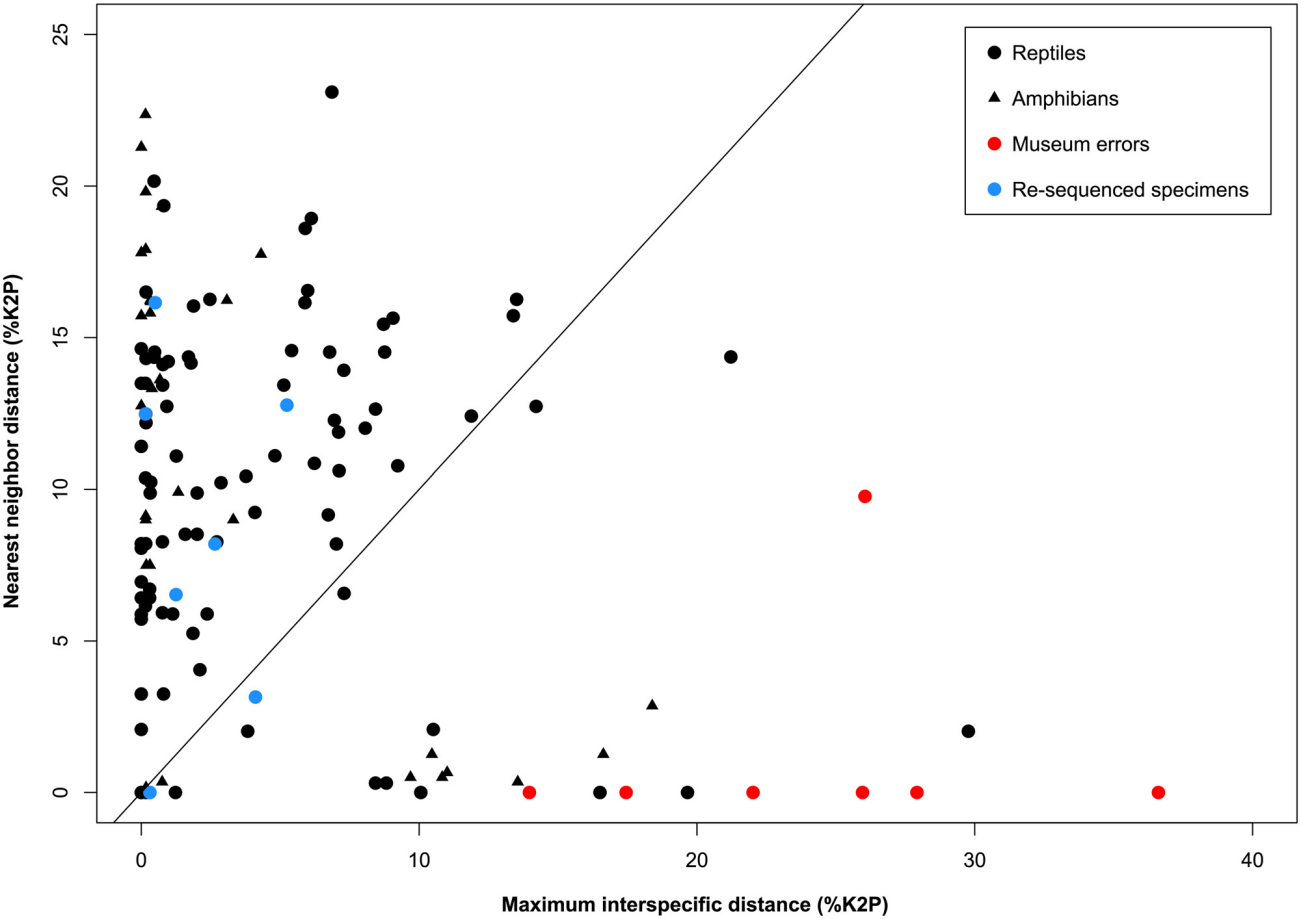
The barcode gap and institutional errors. Comparison of maximum intraspecific sequence divergence with minimum interspecific sequence divergence for amphibians and reptiles. Points above the 1:1 line indicate that a barcode gap is present; points below the line indicate its absence. Points representing museum errors and re-sequenced specimens came from the Royal Ontario Museum.

Using the barcode gap comparison in conjunction with a NJ tree (S1 & S2 Figs), seven specimens from the tissue collection of the Royal Ontario Museum were flagged due to either very low interspecific distances or high intraspecific distances (museum errors in Fig 3). After ensuring that these errors were not due to contamination during DNA extraction, the Royal Ontario Museum sent additional subsamples from the same tissue samples. When these tissues were sequenced, all seven original sequences were found to be incorrect (re-sequenced specimens in Fig 3), with the new sequences clustering within other members of respective species (S1 & S2 Figs). It was subsequently determined that five of the erroneous initial results were due to incorrect labeling of tissue samples while the last case involved mislabeling of the actual sample vial in the Royal Ontario Museum’s tissue collection.

The barcode region in both reptiles and amphibians possessed a high GC content (mean = 43.97%). No significant relationship was observed between family-level nearest neighbor distance and mean GC content in amphibians (R^2^ = 0.01, *p* = 0.40) or reptiles (R^2^ = 0.13, *p* = 0.10). Similarly, there was no significant relationship between nearest neighbor distance and mean GC content in the third codon position (R^2^ = 0.0009, *p* = 0.47 in amphibians; R^2^ = 0.12, *p* = 0.11 in reptiles). No insertions or deletions were observed in the aligned sequences. Substitution saturation occurred at approximately 10–11% sequence divergence for amphibians and at 9–10% for reptiles when considering all three codon positions (S3 Fig).

### Correspondence between BINs and species

**Fig 4:**
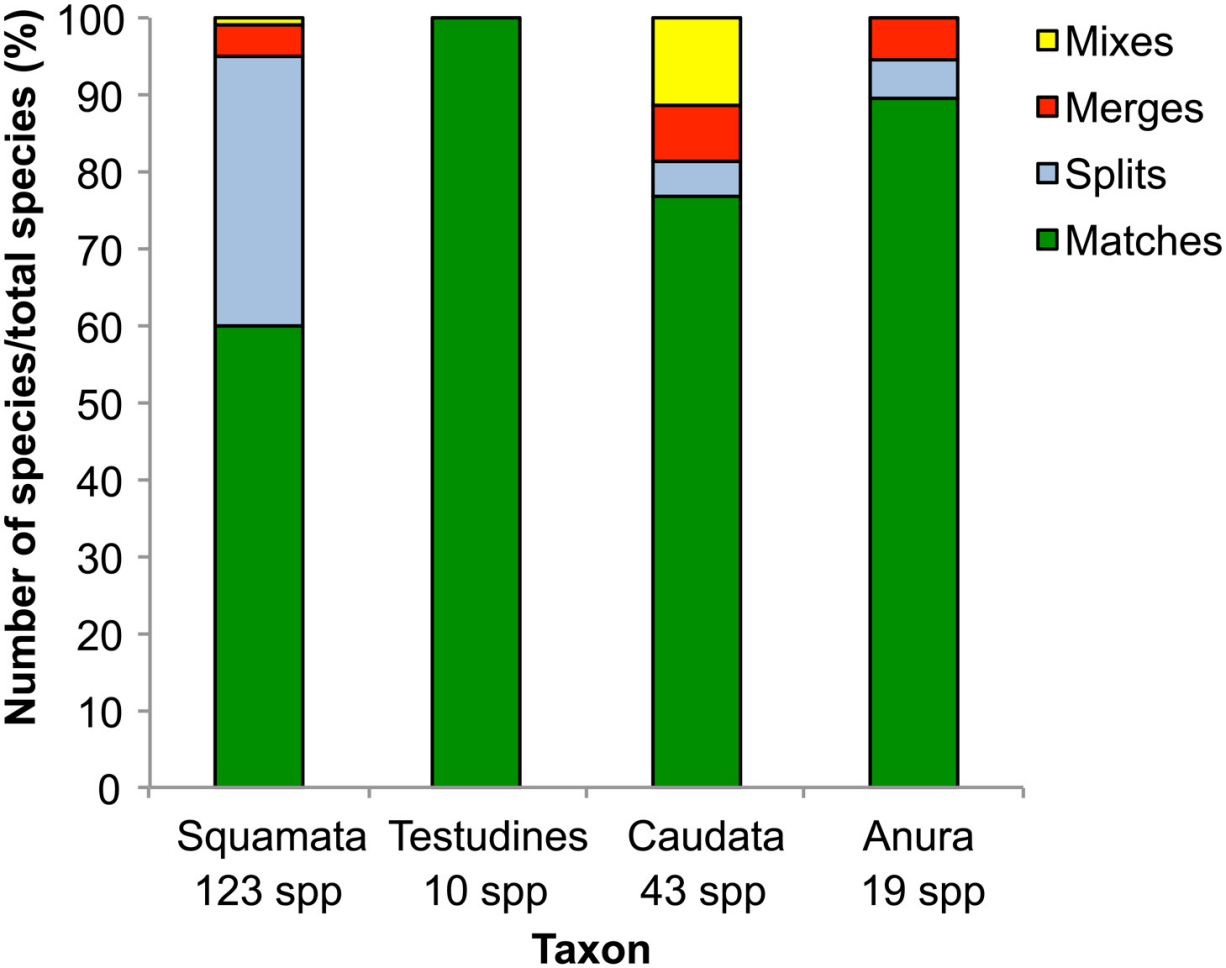
The correspondence between Barcode Index Number (BIN) and species assignment. The number of BIN matches, splits, merges, and mixes for the four orders in this study for barcode-compliant sequences.

There was a correspondence between recognized species boundaries and BIN assignments for 133 of the 195 species (68%), while the discordances largely involved BIN splits. Because the number of splits (one species assigned to two or more BINs) was higher than the number of merges (a single BIN assigned to more than one species), there were 53 more BINs than the corresponding species count for both classes [15] (Fig 4; Table 2). Caudates had the highest number of mixes: merges and splits within the same species (4), while squamates had the highest number of merges (5) and splits (43), the latter of which represented 35% of the total BINs assigned to this order (Table 2). In the reptilian species with BIN splits, sequence divergence was significantly correlated with geographic distance (R^2^ = 0.18, *p*<0.0001). Despite not having a barcode compliant length, 47 specimens (seven species) within both classes were assigned to the correct BIN (range = 359-427bp, mean = 390bp). Additionally, due to the flexible threshold employed by the BIN algorithm, two species of plethodontid salamander with a pairwise distance lower than 2% were assigned to separate BINs (*Desmognathus ocoee* and *D*. *marmoratus*), while five species of reptiles with greater than 2% intraspecific sequence divergence were placed in the BIN corresponding to the other members of their species (*Agkistrodon contortrix*, *Lampropeltis getula*, *Nerodia erythrogaster*, *Storeria dekayi*, and *Thamnophis cyrtopsis*).

**Table 2:** Distance (%K2P) with standard error, Barcode Index Number (BIN), and sequence composition summary for barcode-compliant sequences.

Class	Order	Sequences	Species	BINs	Mean intraspecific distance (%)	Mean congeneric distance (%)	Mean GC content (%)
Amphibia	Anura	24	19	19	2.83(0.75)	16.53(0.11)	44.57(0.41)
	Caudata	100	43	47	1.34(0.04)	16.18(0.01)	40.84(0.31)
Reptilia	Squamata	378	123	172	2.96(0.01)	13.80(0.002)	44.94(0.14)
	Testudines	31	10	10	0.19(0.01)	6.82(1.57)	41.71(0.28)

## Discussion

### Barcode recovery and composition in North American herpetofauna

The “universal” amphibian primer sets [29] were the most effective option for barcode recovery from North American amphibians. Contrary to earlier suggestions that single primer pairs have high failure rates for amphibians [26], these primers consistently recovered sequences across the class. However, in reptiles, our new primers (AmphF2_t1+AmphR3_t1) were the most successful, suggesting that different primer sets should be used for each class to maximize sequence recovery [41].

Saturation of transitions and transversions, considering all three codon positions, occurred at a divergence of 9–11% in both reptiles and amphibians, consistent with previous findings that sequences become saturated in these organisms at approximately 10–13% genetic divergence [3,28,29,42,43]. The lack of insertions and deletions reinforces earlier evidence for their absence in salamanders from the family Hynobiidae [29] and in Korean herpetofauna [44].

### The utility of DNA barcoding in museum collections

The length of sequence recovered decreased for both reptiles and amphibians with increasing specimen age in seven collections, although success rates were consistently high for 30 years after collection, particularly in reptiles. Differences in the recovery of barcode compliant sequences among samples from different institutions likely reflects differential storage conditions (many in ethanol, others frozen). In the case of cryocollections, there was poor documentation of the timing between tissue collection and its transfer into cryostorage.

Although the negative impacts of formalin fixation on DNA sequence recovery are well known (e.g. [16,20,45]), the PCR regime employed in this study led to sequence recovery from 5 of 208 formalin fixed specimens. Further work to improve success from formalin-fixed specimens is justified (e.g. [46]) since so many important specimens, such as holotypes and paratypes, are preserved in this medium [20,47]. Although most of the sequences recovered in this study from formalin-fixed tissues were not barcode compliant, it is important to note that these records can still be very informative for tentative species identification [24,48].

Museum collections have proven a key resource for building DNA barcode reference libraries in earlier studies on insects [21,40], and they could certainly aid the identification of specimens whose morphological diagnosis is difficult or when defining phenotypic characters have deteriorated with age, such as in the North American plethodontid salamanders [42,49]. In fact, this approach detected labeling errors in 2.4% of the specimens from the Royal Ontario Museum—an incidence close to an estimated 5% error rate for museum collections [50].

### Cases of low interspecific divergence and deep intraspecific divergence

The wide variation in sequence divergence for both congeneric and intraspecific comparisons was consistent with prior barcoding studies on reptiles and amphibians that revealed substantial overlap between intra- and interspecific distances [3,25,26,28,29,42,43] (Table 2). Despite this overlap, most (70.4% in reptiles, 87.7% in amphibians) of the intraspecific divergences in this study were less than 3%. The mean intraspecific sequence divergences for both amphibians and reptiles were higher than mean values in birds [51,52], mammals [53,54], and fishes [55,56], mostly due to high maximum distances, and perhaps reflecting overlooked species as indicated by extreme population subdivision and deeply divergent lineages in some taxa [57].

Although BIN clusters did not always correspond to currently recognized species boundaries, exceptions involved previously reported cases of either hybridization or deep divergence. In every instance of BIN split, specimens belonged to a species that showed deep intraspecific divergence, often reflected by population subdivision linked to geographic isolation. The highest incidence of splits were detected in reptiles, whose members belong to old lineages with broad distributions, such as the desert iguana (*Dipsosaurus dorsalis*) and the common chuckwalla (*Sauromalus ater*) [58]. This result is corroborated by the strong correlation between geographic distance and COI divergence, a pattern also observed in other vertebrates (e.g. bats [59]). The species with the highest intraspecific divergence (21.22%) and three BIN splits—the lesser earless lizard (*Holbrookia maculata*)–may include multiple species. Deep mitochondrial divergence was detected in specimens collected in close geographic proximity [60] and reproductive isolation owing to differential mate preference has been suggested [61]. Similarly, splits in amphibians, mostly in salamanders such as *Plethodon caddoensis*, occurred in species with high genetic variability and population subdivision over small geographic ranges [57,62].

BIN mergers occurred in species that are known to hybridize, including the recent divergence and subsequent introgression of *Desmognathus fuscus* and *D*. *ochrophaeus*, as well as the recent speciation of *Pseudacris triseriata* and *P*. *maculata* [63–66]. Additionally, single BIN assignment occurred in four closely allied species pairs of reptiles that exhibit introgression and hybridization: *Aspidoscelis tesselata* and *A*. *neotesselata*, *Sceloporus undulatus* and *S*. *graciosus*, *Plestiodon gilberti* and *P*. *fasciatus*, and *Thamnophis radix* and *T*. *butleri* [67–72].

### Conclusions

This study represents an important first step towards a comprehensive DNA barcode library for North American reptiles and amphibians. By developing a new primer set that facilitates barcode recovery from reptiles, and by confirming the effectiveness of existing primers [29] for amphibians, this study highlights the feasibility of developing barcode coverage for all taxa. The BIN system was effective in recovering established species boundaries in about two thirds of all species, with exceptions involving BIN sharing by species that are known hybridize and BIN splitting in taxa with extensive population subdivision. Consequently, the BIN system can be an effective tool to highlight species suspected of hybridizing, as well as those that may actually represent a species complex. Importantly, in cases where only partial barcodes can be recovered because of DNA degradation, sequences greater than 300bp allow for BIN assignment and usually a reliable identification.

The present results confirm that DNA barcodes have an important role in aiding quality assurance in natural history collections, and provide a simple way to verify that tissues received from cryo-repositories are actually the desired taxon. In groups such as the herpetofauna, where collection permits are often hard to obtain, a method to quickly validate specimen identifications and to detect incorrect database entries is essential. Additionally, DNA barcoding provides a simple approach in the field, where a swab or biopsy sample, ideally paired with accurate locality data and photo vouchers, would be sufficient for preliminary species identifications.

## Supporting Information

S1 Fig Reptilian neighbor-joining tree. Barcode compliant length sequences included, with corresponding collection codes and families. This tree was not used for inferring phylogenetic relationships; it was simply used to visualize distances.
(PDF)

S2 Fig Amphibian neighbor-joining tree. Barcode compliant length sequences included, with corresponding collection codes and families. The only representative for *Rana heckscheri* (collection code: MCZ Herp A-37209) is formalin-fixed. This tree was not used for inferring phylogenetic relationships; it was simply used to visualize distances.
(EPS)

S3 Fig Substitution saturation. The frequency of transitions and transversions with varying levels of sequence divergence (%K2P) considering all three codon positions in (A) amphibians and (B) reptiles.
(PDF)

S1 Table Identification, BOLD process IDs, BOLD sample IDs, and GenBank accession numbers for all successfully sequenced specimens in this study. BOLD sample IDs correspond to institution catalog numbers.
(DOCX)

S2 Table Length of amplicon, DNA dilution factors, success rates, and sample sizes for all specimens (except formalin-fixed). (DOCX)

S3 Table PCR regime details for primers used in this project. (DOCX)

S4 Table Specimens with collection date information included in linear regression analysis. Ages are relative to year of sequencing (2012).
(DOCX)
