# Supplementary figures and images for: Assessing DNA Barcodes for Species Identification in North American Reptiles and Amphibians in Natural History Collections
Source: PLoS One. 2016 Apr 26;11(4):e0154363. doi: 10.1371/journal.pone.0154363 (PMC4846166; doi:10.1371/journal.pone.0154363)

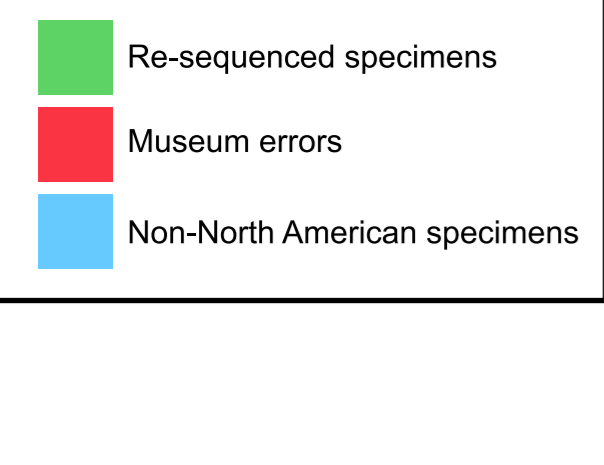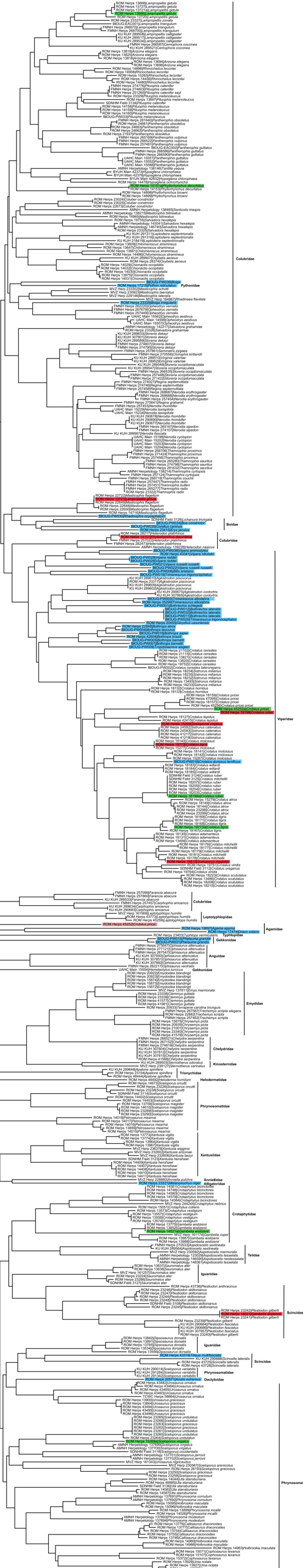

Supplement: S1 Fig — Barcode compliant length sequences included, with corresponding collection codes and families. This tree was not used for inferring phylogenetic relationships; it was simply used to visualize distances. (PDF) [file pone.0154363.s001.pdf]

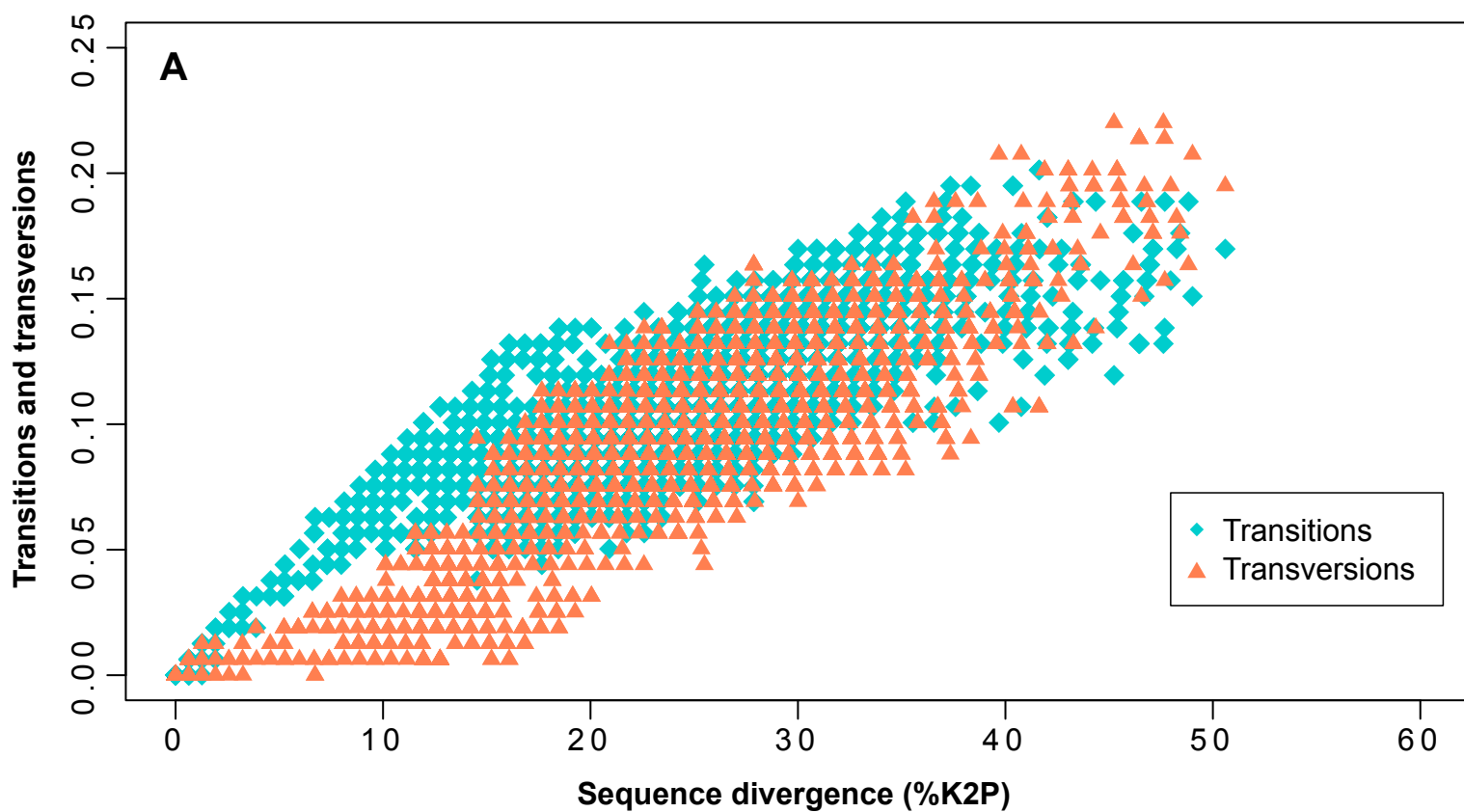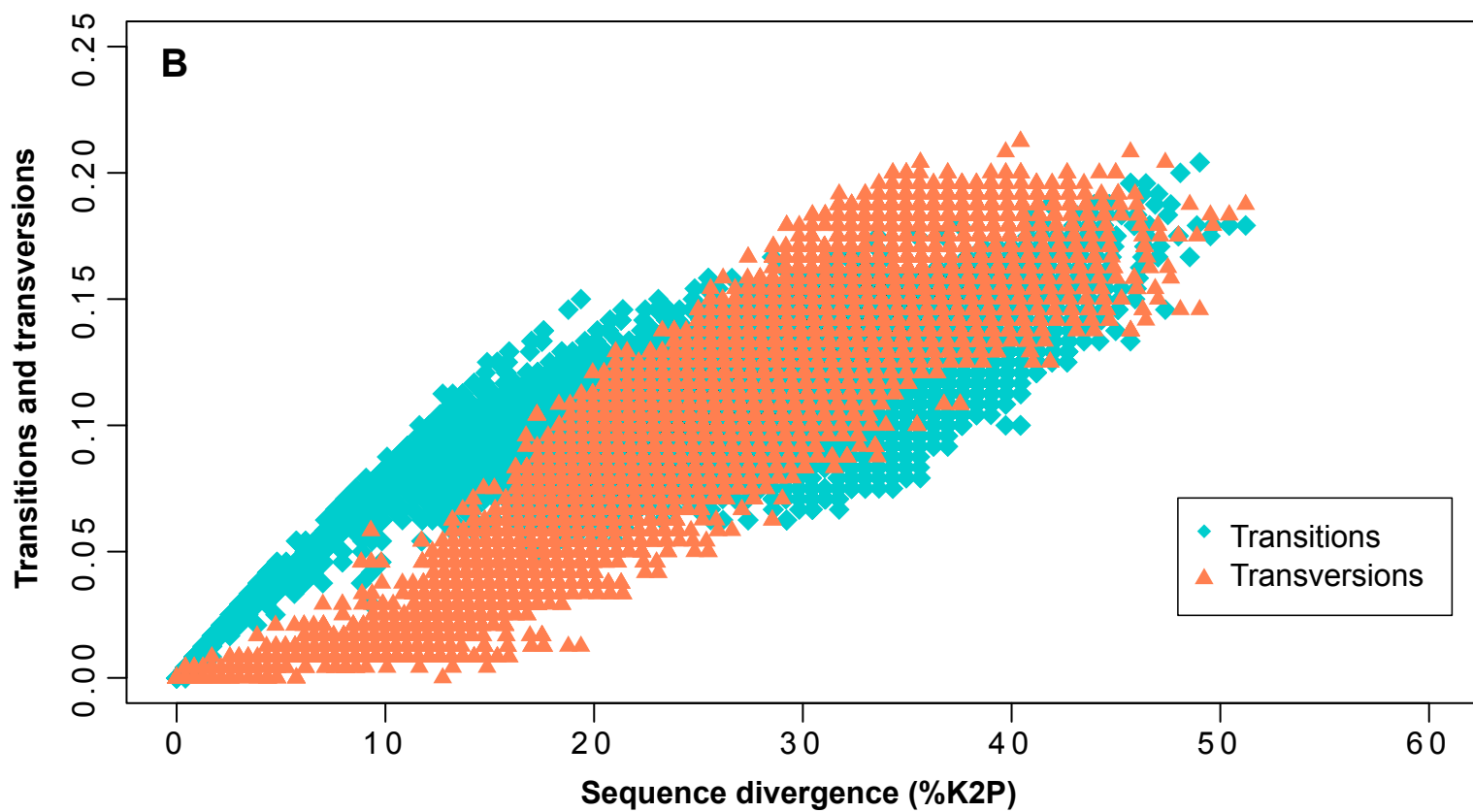

Supplement: S3 Fig — The frequency of transitions and transversions with varying levels of sequence divergence (%K2P) considering all three codon positions in (A) amphibians and (B) reptiles. (PDF) [file pone.0154363.s003.pdf]
