# Supplementary material for: Assessing DNA Barcodes for Species Identification in North American Reptiles and Amphibians in Natural History Collections
Source: PLoS One. 2016 Apr 26;11(4):e0154363. doi: 10.1371/journal.pone.0154363 (PMC4846166; doi:10.1371/journal.pone.0154363)
Supplement: S1 Table — BOLD sample IDs correspond to institution catalog numbers. (DOCX) [file pone.0154363.s004.docx]

**S1 Table. Identification, BOLD process IDs, BOLD sample IDs, and GenBank accession numbers for all successfully sequenced specimens in this study.** BOLD sample IDs correspond to institution catalog numbers.

| **Identification** | **BOLD process ID** | **BOLD sample ID** | **GenBank accession number** |
| --- | --- | --- | --- |
| **Amphibians** |  |  |  |
| *Acris crepitans* | EANAA016-12 | UAHC Main 15984 | KU985874 |
| *Acris crepitans* | EANAA075-12 | FMNH Herps 267572 | KU986159 |
| *Acris crepitans* | EANAA087-12 | FMNH Herps 282523 | KU986210 |
| *Ambystoma annulatum* | EANAA121-12 | ROM Herps 18672 | KU986254 |
| *Ambystoma annulatum* | EANAA122-12 | ROM Herps 18673 | KU985979 |
| *Ambystoma annulatum* | EANAA124-12 | ROM Herps 43781 | KU985678 |
| *Ambystoma californiense* | EANAA125-12 | ROM Herps 20019 | KU986196 |
| *Ambystoma californiense* | EANAA126-12 | ROM Herps 20342 | KU986165 |
| *Ambystoma californiense* | EANAA127-12 | ROM Herps 20343 | KU985632 |
| *Ambystoma californiense* | EANAA128-12 | ROM Herps 20344 | KU986077 |
| *Ambystoma californiense* | EANAA129-12 | ROM Herps 20345 | KU986040 |
| *Ambystoma cingulatum* | EANAA130-12 | ROM Herps 20314 | KU986180 |
| *Ambystoma cingulatum* | EANAA131-12 | ROM Herps 21462 | KU986169 |
| *Ambystoma cingulatum* | EANAA132-12 | ROM Herps 21463 | KU985721 |
| *Ambystoma mabeei* | EANAA204-12 | MVZ Herp 144890 | KU985600 |
| *Ambystoma opacum* | EANAA133-12 | ROM Herps 18784 | KU985606 |
| *Ambystoma opacum* | EANAA134-12 | ROM Herps 18785 | KU986081 |
| *Ambystoma opacum* | EANAA135-12 | ROM Herps 18786 | KU986241 |
| *Ambystoma opacum* | EANAA136-12 | ROM Herps 18787 | KU985732 |
| *Ambystoma opacum* | EANAA137-12 | ROM Herps 18788 | KU986026 |
| *Ambystoma talpoideum* | EANAA139-12 | ROM Herps 21429 | KU985988 |
| *Ambystoma talpoideum* | EANAA140-12 | ROM Herps 21430 | KU986170 |
| *Ambystoma talpoideum* | EANAA141-12 | ROM Herps 21431 | KU986131 |
| *Ambystoma talpoideum* | EANAA142-12 | ROM Herps 21432 | KU985959 |
| *Ambystoma texanum* | EANAA143-12 | ROM Herps 18325 | KU985675 |
| *Ambystoma texanum* | EANAA144-12 | ROM Herps 18326 | KU986051 |
| *Ambystoma texanum* | EANAA145-12 | ROM Herps 18327 | KU985628 |
| *Ambystoma texanum* | EANAA146-12 | ROM Herps 18328 | KU985602 |
| *Ambystoma texanum* | EANAA147-12 | ROM Herps 18329 | KU985559 |
| *Ambystoma tigrinum* | EANAA148-12 | ROM Herps 16010 | KU985809 |
| *Ambystoma tigrinum* | EANAA149-12 | ROM Herps 16009 | KU986072 |
| *Ambystoma tigrinum* | EANAA150-12 | ROM Herps 16011 | KU986212 |
| *Ambystoma tigrinum* | EANAA151-12 | ROM Herps 16012 | KU986050 |
| *Ambystoma tigrinum* | EANAA152-12 | ROM Herps 16013a | KU985967 |
| *Amphiuma means* | EANAA194-12 | AMNH Herpetology 168430 | KU986280 |
| *Aneides ferreus* | EANAA153-12 | ROM Herps 18768 | KU985575 |
| *Aneides ferreus* | EANAA154-12 | ROM Herps 18769 | KU985975 |
| *Aneides ferreus* | EANAA155-12 | ROM Herps 21575 | KU986100 |
| *Aneides ferreus* | EANAA156-12 | ROM Herps 21576 | KU985816 |
| *Aneides ferreus* | EANAA157-12 | ROM Herps 21577 | KU985743 |
| *Aneides flavipunctatus* | EANAA158-12 | ROM Herps 18770 | KU985901 |
| *Ascaphus montanus* | EANAA239-12 | MVZ Herp 231861 | KU985903 |
| *Batrachoseps diabolicus* | EANAA234-12 | MVZ Herp 156543 | KU985771 |
| *Batrachoseps gabrieli* | EANAA235-12 | MVZ Herp 237193 | KU985855 |
| *Batrachoseps gavilanensis* | EANAA207-12 | MVZ Herp 154716 | KU985803 |
| *Batrachoseps gregarius* | EANAA208-12 | MVZ Herp 224560 | KU985971 |
| *Batrachoseps kawia* | EANAA236-12 | MVZ Herp 178630 | KU986078 |
| *Batrachoseps major* | EANAA210-12 | MVZ Herp 225665 | KU985735 |
| *Batrachoseps pacificus* | EANAA212-12 | MVZ Herp 157401 | KU986125 |
| *Batrachoseps regius* | EANAA213-12 | MVZ Herp 224804 | KU985736 |
| *Batrachoseps robustus* | EANAA237-12 | MVZ Herp 222942 | KU986099 |
| *Batrachoseps simatus* | EANAA214-12 | MVZ Herp 156460 | KU985846 |
| *Bufo boreas* | EANAA003-12 | SDNHM Field 3089 | KU986258 |
| *Bufo cognatus* | EANAA241-12 | MVZ Herp 245866 | KU985788 |
| *Bufo quercicus* | EANAA195-12 | AMNH Herpetology 168431 | KU986042 |
| *Bufo speciosus* | EANAA244-12 | MVZ Herp 226837 | KU985872 |
| *Bufo woodhousii* | EANAA093-12 | KU KUH 224658 | KU986049 |
| *Bufo woodhousii* | EANAA094-12 | KU KUH 290544 | KU986010 |
| *Bufo woodhousii* | EANAA095-12 | KU KUH 290549 | KU985902 |
| *Cryptobranchus alleganiensis* | EANAA159-12 | ROM Herps 22332 | KU985595 |
| *Cryptobranchus alleganiensis* | EANAA160-12 | ROM Herps 22333 | KU985766 |
| *Desmognathus aeneus* | EANAA019-12 | UAHC Main 16041 | KU985866 |
| *Desmognathus brimleyorum* | EANAA216-12 | MVZ Herp 215258 | KU986036 |
| *Desmognathus conanti* | EANAA022-12 | UAHC Main 16088 | KU986277 |
| *Desmognathus conanti* | EANAA023-12 | UAHC Main 15912 | KU985932 |
| *Desmognathus fuscus* | EANAA024-12 | UAHC Main 14535 | KU986127 |
| *Desmognathus fuscus* | EANAA025-12 | UAHC Main 15019 | KU985621 |
| *Desmognathus fuscus* | EANAA026-12 | UAHC Main 15380 | KU985661 |
| *Desmognathus imitator* | EANAA217-12 | MVZ Herp 219994 | KU986147 |
| *Desmognathus marmoratus* | EANAA027-12 | UAHC Main 16128 | KU986191 |
| *Desmognathus marmoratus* | EANAA028-12 | UAHC Main 16123 | KU986016 |
| *Desmognathus marmoratus* | EANAA029-12 | UAHC Main 16125 | KU986200 |
| *Desmognathus monticola* | EANAA030-12 | UAHC Main 16026 | KU985843 |
| *Desmognathus monticola* | EANAA031-12 | UAHC Main 15936 | KU985839 |
| *Desmognathus ochrophaeus* | EANAA161-12 | ROM Herps 15974 | KU986025 |
| *Desmognathus ochrophaeus* | EANAA162-12 | ROM Herps 15975 | KU985783 |
| *Desmognathus ochrophaeus* | EANAA163-12 | ROM Herps 15976 | KU985710 |
| *Desmognathus ochrophaeus* | EANAA164-12 | ROM Herps 21480 | KU986074 |
| *Desmognathus ochrophaeus* | EANAA165-12 | ROM Herps 21481 | KU985633 |
| *Desmognathus ocoee* | EANAA032-12 | UAHC Main 15032 | KU986061 |
| *Desmognathus ocoee* | EANAA033-12 | UAHC Main 15035 | KU985983 |
| *Desmognathus quadramaculatus* | EANAA034-12 | UAHC Main 16121 | KU985708 |
| *Desmognathus quadramaculatus* | EANAA035-12 | UAHC Main 16124 | KU985833 |
| *Desmognathus quadramaculatus* | EANAA036-12 | UAHC Main 16120 | KU985669 |
| *Desmognathus santeetlah* | EANAA218-12 | MVZ Herp 222604 | KU985726 |
| *Desmognathus wrighti* | EANAA037-12 | UAHC Main 16063 | KU986239 |
| *Desmognathus wrighti* | EANAA038-12 | UAHC Main 16074 | KU985850 |
| *Desmognathus wrighti* | EANAA039-12 | UAHC Main 16070 | KU985585 |
| *Dicamptodon aterrimus* | EANAA205-12 | MVZ Herp 187986 | KU985689 |
| *Dicamptodon ensatus* | EANAA166-12 | ROM Herps 18771 | KU985656 |
| *Dicamptodon ensatus* | EANAA167-12 | ROM Herps 18772 | KU985838 |
| *Dicamptodon ensatus* | EANAA168-12 | ROM Herps 18764 | KU985655 |
| *Dicamptodon ensatus* | EANAA169-12 | ROM Herps 18766 | KU985936 |
| *Dicamptodon ensatus* | EANAA170-12 | ROM Herps 18767 | KU986281 |
| *Ensatina klauberi* | EANAA001-12 | SDNHM Field 3110 | KU986110 |
| *Eurycea cirrigera* | EANAA078-12 | FMNH Herps 278447 | KU985762 |
| *Eurycea cirrigera* | EANAA079-12 | FMNH Herps 278801 | KU986096 |
| *Eurycea guttolineata* | EANAA040-12 | UAHC Main 16040 | KU986184 |
| *Eurycea guttolineata* | EANAA041-12 | UAHC Main 15959 | KU985716 |
| *Eurycea guttolineata* | EANAA042-12 | UAHC Main 15483 | KU986269 |
| *Eurycea longicauda* | EANAA219-12 | MVZ Herp 219995 | KU986058 |
| *Eurycea multiplicata* | EANAA220-12 | MVZ Herp 145024 | KU985734 |
| *Gastrophryne carolinensis* | EANAA096-12 | KU KUH 289624 | KU985647 |
| *Gyrinophilus porphyriticus* | EANAA171-12 | ROM Herps 15980 | KU985948 |
| *Gyrinophilus porphyriticus* | EANAA172-12 | ROM Herps 15986 | KU985968 |
| *Gyrinophilus porphyriticus* | EANAA173-12 | ROM Herps 15987 | KU985608 |
| *Gyrinophilus porphyriticus* | EANAA174-12 | ROM Herps 16013 | KU985642 |
| *Gyrinophilus porphyriticus* | EANAA175-12 | ROM Herps 16036 | KU985794 |
| *Hemidactylium scutatum* | EANAA043-12 | UAHC Main 15714 | KU985999 |
| *Hemidactylium scutatum* | EANAA044-12 | UAHC Main 16134 | KU985696 |
| *Hydromantes platycephalus* | EANAA221-12 | MVZ Herp 249937 | KU985943 |
| *Hyla avivoca* | EANAA098-12 | KU KUH 207348 | KU985880 |
| *Hyla avivoca* | EANAA196-12 | AMNH Herpetology 168424 | KU985972 |
| *Hyla chrysoscelis* | EANAA100-12 | KU KUH 207352 | KU985886 |
| *Hyla chrysoscelis* | EANAA101-12 | KU KUH 207353 | KU985964 |
| *Hyla chrysoscelis* | EANAA102-12 | KU KUH 207354 | KU985564 |
| *Hyla cinerea* | EANAA103-12 | KU KUH 207358 | KU985597 |
| *Hyla cinerea* | EANAA104-12 | KU KUH 207359 | KU986162 |
| *Hyla cinerea* | EANAA105-12 | KU KUH 207360 | KU985703 |
| *Hyla femoralis* | EANAA197-12 | AMNH Herpetology 168426 | KU986228 |
| *Hyla gratiosa* | EANAA046-12 | UAHC Main 14739 | KU985792 |
| *Hyla wrightorum* | EANAA296-13 | USNM 245994 | KU985589 |
| *Hypopachus variolosus* | EANAA106-12 | KU KUH 289842 | KU986205 |
| *Hypopachus variolosus* | EANAA107-12 | KU KUH 291269 | KU985729 |
| *Hypopachus variolosus* | EANAA108-12 | KU KUH 291270 | KU985750 |
| *Incilius alvarius* | EANAA240-12 | MVZ Herp 132966 | KU986203 |
| *Incilius nebulifer* | EANAA243-12 | MVZ Herp 145250 | KU986174 |
| *Necturus alabamensis* | EANAA230-12 | MVZ Herp 187705 | KU985890 |
| *Necturus beyeri* | EANAA049-12 | UAHC Main 15628 | KU986247 |
| *Necturus lewisi* | EANAA231-12 | MVZ Herp 501291 | KU985919 |
| *Necturus maculosus* | EANAA080-12 | FMNH Herps 261585 | KU985645 |
| *Necturus maculosus* | EANAA081-12 | FMNH Herps 261595 | KU985578 |
| *Necturus maculosus* | EANAA178-12 | ROM Herps 23138 | KU985984 |
| *Necturus punctatus* | EANAA232-12 | MVZ Herp 187708 | KU985769 |
| *Notophthalmus viridescens* | EANAA082-12 | FMNH Herps 267571 | KU985820 |
| *Plethodon albagula* | EANAA109-12 | KU KUH 218912 | KU985966 |
| *Plethodon albagula* | EANAA110-12 | KU KUH 218913 | KU986279 |
| *Plethodon caddoensis* | EANAA112-12 | KU KUH 218930 | KU985859 |
| *Plethodon caddoensis* | EANAA113-12 | KU KUH 218931 | KU986019 |
| *Plethodon dunni* | EANAA238-12 | MVZ Herp 218082 | KU986158 |
| *Plethodon fourchensis* | EANAA222-12 | MVZ Herp 215255 | KU986243 |
| *Plethodon glutinosus* | EANAA179-12 | ROM Herps 20427 | KU985910 |
| *Plethodon glutinosus* | EANAA180-12 | ROM Herps 21556 | KU985638 |
| *Plethodon glutinosus* | EANAA181-12 | ROM Herps 21562 | KU986056 |
| *Plethodon glutinosus* | EANAA182-12 | ROM Herps 21563 | KU986069 |
| *Plethodon glutinosus* | EANAA183-12 | ROM Herps 21564 | KU986027 |
| *Plethodon grobmani* | EANAA054-12 | UAHC Main 15792 | KU986154 |
| *Plethodon grobmani* | EANAA055-12 | UAHC Main 15794 | KU986107 |
| *Plethodon hoffmani* | EANAA223-12 | MVZ Herp 137290 | KU985922 |
| *Plethodon jordani* | EANAA225-12 | MVZ Herp 137293 | KU986146 |
| *Plethodon mississippi* | EANAA057-12 | UAHC Main 16087 | KU985713 |
| *Plethodon mississippi* | EANAA058-12 | UAHC Main 15848 | KU986176 |
| *Plethodon montanus* | EANAA059-12 | UAHC Main 15761 | KU986011 |
| *Plethodon montanus* | EANAA060-12 | UAHC Main 15779 | KU985917 |
| *Plethodon montanus* | EANAA061-12 | UAHC Main 15815 | KU985900 |
| *Plethodon ouachitae* | EANAA114-12 | KU KUH 218917 | KU986090 |
| *Plethodon ouachitae* | EANAA115-12 | KU KUH 218918 | KU986006 |
| *Plethodon serratus* | EANAA062-12 | UAHC Main 16006 | KU985905 |
| *Plethodon serratus* | EANAA063-12 | UAHC Main 15979 | KU985619 |
| *Plethodon serratus* | EANAA064-12 | UAHC Main 15971 | KU985828 |
| *Plethodon teyahalee* | EANAA226-12 | MVZ Herp 206570 | KU985764 |
| *Plethodon vehiculum* | EANAA184-12 | ROM Herps 21534 | KU986190 |
| *Plethodon vehiculum* | EANAA185-12 | ROM Herps 21535 | KU985748 |
| *Plethodon vehiculum* | EANAA186-12 | ROM Herps 21536 | KU985649 |
| *Plethodon vehiculum* | EANAA187-12 | ROM Herps 21537 | KU985882 |
| *Plethodon vehiculum* | EANAA188-12 | ROM Herps 21538 | KU985740 |
| *Plethodon websteri* | EANAA065-12 | UAHC Main 15947 | KU986234 |
| *Plethodon yonahlossee* | EANAA228-12 | MVZ Herp 225739 | KU985784 |
| *Pseudacris brachyphona* | EANAA068-12 | UAHC Main 14996 | KU985927 |
| *Pseudacris brachyphona* | EANAA069-12 | UAHC Main 14997 | KU986231 |
| *Pseudacris brachyphona* | EANAA070-12 | UAHC Main 14998 | KU985587 |
| *Pseudacris cadaverina* | EANAA245-12 | MVZ Herp 145383 | KU985837 |
| *Pseudacris maculata* | EANAA005-12 | BIOUG-EAC004 | KU986064 |
| *Pseudacris maculata* | EANAA006-12 | BIOUG-EAC005 | KU985712 |
| *Pseudacris maculata* | EANAA007-12 | BIOUG-EAC006 | KU985634 |
| *Pseudacris ocularis* | EANAA198-12 | AMNH Herpetology 168474 | KU985761 |
| *Pseudacris ocularis* | EANAA199-12 | AMNH Herpetology 168473 | KU986132 |
| *Pseudacris triseriata* | EANAA083-12 | FMNH Herps 279400 | KU985840 |
| *Pseudacris triseriata* | EANAA085-12 | FMNH Herps 271428 | KU985780 |
| *Pseudacris triseriata* | EANAA088-12 | FMNH Herps 281637 | KU985707 |
| *Pseudotriton montanus* | EANAA229-12 | MVZ Herp 501304 | KU986117 |
| *Pseudotriton ruber* | EANAA072-12 | UAHC Main 15969 | KU985728 |
| *Rana areolata* | EANAA250-12 | MVZ Herp 145472 | KU986008 |
| *Rana blairi* | EANAA251-12 | MVZ Herp 240136 | KU985826 |
| *Rana cascadae* | EANAA252-12 | MVZ Herp 148969 | KU985818 |
| *Rana draytonii* | EANAA253-12 | MVZ Herp 229366 | KU985596 |
| *Rana heckscheri* | EANAA274-12 | MCZ Herp A-37209 | KU985912 |
| *Rana luteiventris* | EANAA254-12 | MVZ Herp 137417 | KU985757 |
| *Rana muscosa* | EANAA255-12 | MVZ Herp 226112 | KU985709 |
| *Rana sierrae* | EANAA257-12 | MVZ Herp 180163 | KU985650 |
| *Rana sylvatica* | EANAA010-12 | BIOUG-EAC009 | KU986227 |
| *Rana sylvatica* | EANAA011-12 | BIOUG-EAC010 | KU985685 |
| *Rana sylvatica* | EANAA012-12 | BIOUG-EAC011 | KU985561 |
| *Rana sylvatica* | EANAA013-12 | BIOUG-EAC012 | KU986084 |
| *Rana virgatipes* | EANAA256-12 | MVZ Herp 137434 | KU985768 |
| *Rhinophrynus dorsalis* | EANAA116-12 | KU KUH 207511 | KU986215 |
| *Rhyacotriton olympicus* | EANAA189-12 | ROM Herps 20416 | KU986021 |
| *Rhyacotriton olympicus* | EANAA190-12 | ROM Herps 20417 | KU986249 |
| *Rhyacotriton olympicus* | EANAA191-12 | ROM Herps 20418 | KU985563 |
| *Rhyacotriton olympicus* | EANAA192-12 | ROM Herps 21581 | KU985758 |
| *Rhyacotriton olympicus* | EANAA193-12 | ROM Herps 21582 | KU985892 |
| *Rhyacotriton variegatus* | EANAA387-13 | USNM 193232 | KU986123 |
| *Scaphiopus couchii* | EANAA247-12 | MVZ Herp 145186 | KU986024 |
| *Scaphiopus holbrookii* | EANAA084-12 | FMNH Herps 259980 | KU985774 |
| *Siren intermedia* | EANAA120-12 | ROM Herps 20351 | KU985926 |
| *Smilisca fodiens* | EANAA246-12 | MVZ Herp 133003 | KU985711 |
| *Spea bombifrons* | EANAA248-12 | MVZ Herp 241592 | KU985593 |
| *Spea hammondii* | EANAA118-12 | KU KUH 209987 | KU986102 |
| *Spea intermontana* | EANAA119-12 | KU KUH 209988 | KU986138 |
| *Spea multiplicata* | EANAA249-12 | MVZ Herp 234197 | KU986202 |
| *Taricha torosa* | EANAA233-12 | MVZ Herp 236243 | KU986086 |
| **Reptiles** |  |  |  |
| *Agkistrodon contortrix* | EANAH566-12 | KU KUH 290675 | KU985668 |
| *Agkistrodon contortrix* | EANAH587-12 | KU KUH 307865 | KU985795 |
| *Agkistrodon contortrix* | EANAH767-12 | ROM Herps 18230 | KU985836 |
| *Agkistrodon piscivorus* | EANAH558-12 | KU KUH 289602 | KU986126 |
| *Agkistrodon piscivorus* | EANAH559-12 | KU KUH 289609 | KU985861 |
| *Agkistrodon piscivorus* | EANAH560-12 | KU KUH 289610 | KU985592 |
| *Agkistrodon piscivorus* | EANAH838-12 | ROM Herps 23317 | KU986244 |
| *Anniella pulchra* | EANAH930-12 | MVZ Herp 228860 | KU985941 |
| *Apalone ferox* | EANAH1048-13 | BIOUG-EAC018 | KU985614 |
| *Apalone ferox* | EANAH1051-13 | BIOUG-EAC021 | KU985742 |
| *Apalone ferox* | EANAH1054-13 | BIOUG-EAC024 | KU985920 |
| *Apalone ferox* | EANAH1055-13 | BIOUG-EAC025 | KU985913 |
| *Apalone ferox* | EANAH1056-13 | BIOUG-EAC026 | KU986257 |
| *Apalone ferox* | EANAH1058-13 | BIOUG-EAC028 | KU986038 |
| *Apalone ferox* | EANAH1062-13 | BIOUG-EAC032 | KU985688 |
| *Apalone ferox* | EANAH1064-13 | BIOUG-EAC034 | KU985706 |
| *Apalone ferox* | EANAH1065-13 | BIOUG-EAC035 | KU985704 |
| *Apalone ferox* | EANAH1066-13 | BIOUG-EAC036 | KU986175 |
| *Apalone ferox* | EANAH1067-13 | BIOUG-EAC037 | KU986047 |
| *Apalone ferox* | EANAH1069-13 | BIOUG-EAC039 | KU985844 |
| *Apalone ferox* | EANAH1070-13 | BIOUG-EAC040 | KU986062 |
| *Apalone ferox* | EANAH1071-13 | BIOUG-EAC041 | KU986148 |
| *Apalone ferox* | EANAH1072-13 | BIOUG-EAC042 | KU986033 |
| *Apalone ferox* | EANAH1073-13 | BIOUG-EAC043 | KU985884 |
| *Apalone ferox* | EANAH1074-13 | BIOUG-EAC044 | KU986017 |
| *Apalone ferox* | EANAH1080-13 | BIOUG-EAC050 | KU985751 |
| *Apalone ferox* | EANAH1086-13 | BIOUG-EAC056 | KU985697 |
| *Apalone ferox* | EANAH1087-13 | BIOUG-EAC057 | KU985857 |
| *Apalone spinifera* | EANAH1047-13 | BIOUG-EAC017 | KU986115 |
| *Apalone spinifera* | EANAH1049-13 | BIOUG-EAC019 | KU986001 |
| *Apalone spinifera* | EANAH1050-13 | BIOUG-EAC020 | KU985746 |
| *Apalone spinifera* | EANAH1052-13 | BIOUG-EAC022 | KU985817 |
| *Apalone spinifera* | EANAH1053-13 | BIOUG-EAC023 | KU985834 |
| *Apalone spinifera* | EANAH1057-13 | BIOUG-EAC027 | KU986101 |
| *Apalone spinifera* | EANAH1060-13 | BIOUG-EAC030 | KU985560 |
| *Apalone spinifera* | EANAH1061-13 | BIOUG-EAC031 | KU986089 |
| *Apalone spinifera* | EANAH1068-13 | BIOUG-EAC038 | KU986142 |
| *Apalone spinifera* | EANAH1075-13 | BIOUG-EAC045 | KU985776 |
| *Apalone spinifera* | EANAH1076-13 | BIOUG-EAC046 | KU985660 |
| *Apalone spinifera* | EANAH1077-13 | BIOUG-EAC047 | KU985923 |
| *Apalone spinifera* | EANAH1078-13 | BIOUG-EAC048 | KU986261 |
| *Apalone spinifera* | EANAH1079-13 | BIOUG-EAC049 | KU985835 |
| *Apalone spinifera* | EANAH1081-13 | BIOUG-EAC051 | KU985626 |
| *Apalone spinifera* | EANAH1082-13 | BIOUG-EAC052 | KU985767 |
| *Apalone spinifera* | EANAH1083-13 | BIOUG-EAC053 | KU985798 |
| *Apalone spinifera* | EANAH1084-13 | BIOUG-EAC054 | KU985723 |
| *Apalone spinifera* | EANAH1085-13 | BIOUG-EAC055 | KU985986 |
| *Apalone spinifera* | EANAH1088-13 | BIOUG-EAC058 | KU986045 |
| *Apalone spinifera* | EANAH563-12 | KU KUH 289648 | KU986251 |
| *Apalone spinifera* | EANAH803-12 | ROM Herps 23154 | KU985800 |
| *Apalone spinifera* | EANAH899-12 | ROM Herps 46444 | KU986130 |
| *Arizona elegans* | EANAH620-12 | ROM Herps 13694 | KU986177 |
| *Arizona elegans* | EANAH621-12 | ROM Herps 13696 | KU986204 |
| *Arizona elegans* | EANAH640-12 | ROM Herps 13818 | KU986091 |
| *Arizona elegans* | EANAH641-12 | ROM Herps 13819 | KU985851 |
| *Arizona elegans* | EANAH644-12 | ROM Herps 13825 | KU985570 |
| *Aspidoscelis marmorata* | EANAH956-12 | MVZ Herp 230592 | KU985775 |
| *Aspidoscelis neotesselata* | EANAH412-12 | AMNH Herpetology 146594 | KU986097 |
| *Aspidoscelis neotesselata* | EANAH413-12 | AMNH Herpetology 146595 | KU985782 |
| *Aspidoscelis sexlineata* | EANAH394-12 | AMNH Herpetology 119490 | KU986080 |
| *Aspidoscelis sexlineata* | EANAH524-12 | FMNH Herps 270533 | KU985574 |
| *Aspidoscelis sexlineata* | EANAH555-12 | KU KUH 289564 | KU986209 |
| *Aspidoscelis tesselata* | EANAH395-12 | AMNH Herpetology 123029 | KU986116 |
| *Aspidoscelis tesselata* | EANAH416-12 | AMNH Herpetology 148361 | KU985929 |
| *Callisaurus draconoides* | EANAH627-12 | ROM Herps 13746 | KU985683 |
| *Callisaurus draconoides* | EANAH628-12 | ROM Herps 13754 | KU986098 |
| *Callisaurus draconoides* | EANAH629-12 | ROM Herps 13755 | KU985875 |
| *Callisaurus draconoides* | EANAH635-12 | ROM Herps 13776 | KU986119 |
| *Callisaurus draconoides* | EANAH636-12 | ROM Herps 13777 | KU985860 |
| *Carphophis amoenus* | EANAH483-12 | FMNH Herps 257457 | KU985611 |
| *Carphophis amoenus* | EANAH562-12 | KU KUH 289634 | KU986263 |
| *Carphophis amoenus* | EANAH569-12 | KU KUH 290683 | KU986237 |
| *Carphophis vermis* | EANAH932-12 | MVZ Herp 137556 | KU986240 |
| *Cemophora coccinea* | EANAH500-12 | FMNH Herps 266587 | KU985700 |
| *Cemophora coccinea* | EANAH549-12 | KU KUH 289521 | KU985680 |
| *Chelydra serpentina* | EANAH496-12 | FMNH Herps 266521 | KU985648 |
| *Chelydra serpentina* | EANAH509-12 | FMNH Herps 267152 | KU985806 |
| *Chelydra serpentina* | EANAH536-12 | FMNH Herps 274819 | KU985719 |
| *Chelydra serpentina* | EANAH580-12 | KU KUH 307804 | KU985815 |
| *Chelydra serpentina* | EANAH581-12 | KU KUH 307812 | KU985770 |
| *Chelydra serpentina* | EANAH582-12 | KU KUH 307815 | KU986272 |
| *Chelydra serpentina* | EANAH868-12 | ROM Herps 41588 | KU985607 |
| *Chilomeniscus stramineus* | EANAH612-12 | ROM Herps 13609 | KU985641 |
| *Chilomeniscus stramineus* | EANAH615-12 | ROM Herps 13647 | KU985637 |
| *Chilomeniscus stramineus* | EANAH616-12 | ROM Herps 13661 | KU985663 |
| *Chilomeniscus stramineus* | EANAH695-12 | ROM Herps 14486 | KU985995 |
| *Chionactis occipitalis* | EANAH649-12 | ROM Herps 13875 | KU986137 |
| *Chionactis occipitalis* | EANAH669-12 | ROM Herps 14029 | KU985889 |
| *Chionactis occipitalis* | EANAH670-12 | ROM Herps 14030 | KU985841 |
| *Chionactis occipitalis* | EANAH671-12 | ROM Herps 14031 | KU986168 |
| *Chionactis occipitalis* | EANAH672-12 | ROM Herps 14032 | KU986106 |
| *Chrysemys picta* | EANAH731-12 | ROM Herps 15875 | KU985617 |
| *Chrysemys picta* | EANAH787-12 | ROM Herps 20935 | KU985777 |
| *Chrysemys picta* | EANAH788-12 | ROM Herps 21001 | KU985772 |
| *Chrysemys picta* | EANAH848-12 | ROM Herps 23340 | KU986087 |
| *Chrysemys picta* | EANAH867-12 | ROM Herps 41579 | KU986274 |
| *Clemmys guttata* | EANAH846-12 | ROM Herps 23338 | KU986128 |
| *Clemmys guttata* | EANAH847-12 | ROM Herps 23339 | KU985630 |
| *Clemmys guttata* | EANAH866-12 | ROM Herps 41577 | KU985699 |
| *Clemmys guttata* | EANAH869-12 | ROM Herps 41981 | KU985867 |
| *Clemmys muhlenbergii* | EANAH835-12 | ROM Herps 23296 | KU985629 |
| *Clonophis kirtlandii* | EANAH525-12 | FMNH Herps 270556 | KU986171 |
| *Coleonyx brevis* | EANAH884-12 | ROM Herps 43680 | KU986253 |
| *Coleonyx brevis* | EANAH885-12 | ROM Herps 43681 | KU986113 |
| *Coleonyx brevis* | EANAH886-12 | ROM Herps 43682 | KU985664 |
| *Coleonyx brevis* | EANAH887-12 | ROM Herps 43684 | KU985567 |
| *Coleonyx switaki* | EANAH596-12 | ROM Herps 13291 | KU985730 |
| *Coleonyx switaki* | EANAH804-12 | ROM Herps 23163 | KU985904 |
| *Coleonyx switaki* | EANAH805-12 | ROM Herps 23164 | KU985876 |
| *Coleonyx switaki* | EANAH806-12 | ROM Herps 23165 | KU985738 |
| *Coleonyx switaki* | EANAH807-12 | ROM Herps 23166 | KU986108 |
| *Coleonyx variegatus* | EANAH638-12 | ROM Herps 13792 | KU986163 |
| *Coleonyx variegatus* | EANAH639-12 | ROM Herps 13796 | KU985741 |
| *Coleonyx variegatus* | EANAH674-12 | ROM Herps 14071 | KU986211 |
| *Coleonyx variegatus* | EANAH675-12 | ROM Herps 14079 | KU986188 |
| *Coleonyx variegatus* | EANAH676-12 | ROM Herps 14083 | KU986111 |
| *Coleonyx variegatus* | EANAH691-12 | ROM Herps 14465 | KU985869 |
| *Coleonyx variegatus* | EANAH692-12 | ROM Herps 14466 | KU985623 |
| *Coleonyx variegatus* | EANAH862-12 | ROM Herps 38480 | KU985582 |
| *Coleonyx variegatus* | EANAH888-12 | ROM Herps 43697 | KU985677 |
| *Coleonyx variegatus* | EANAH889-12 | ROM Herps 43699 | KU985868 |
| *Coleonyx variegatus* | EANAH890-12 | ROM Herps 43700 | KU985636 |
| *Coleonyx variegatus* | EANAH891-12 | ROM Herps 43704 | KU985754 |
| *Coluber constrictor* | EANAH795-12 | ROM Herps 22672 | KU985813 |
| *Coluber constrictor* | EANAH796-12 | ROM Herps 22673 | KU985863 |
| *Coluber constrictor* | EANAH801-12 | ROM Herps 23024 | KU986055 |
| *Coluber constrictor* | EANAH802-12 | ROM Herps 23025 | KU986094 |
| *Contia tenuis* | EANAH933-12 | MVZ Herp 257257 | KU986070 |
| *Cophosaurus texanus* | EANAH717-12 | ROM Herps 15071 | KU985977 |
| *Cophosaurus texanus* | EANAH720-12 | ROM Herps 15107 | KU985976 |
| *Cophosaurus texanus* | EANAH727-12 | ROM Herps 15372 | KU986022 |
| *Crotalus adamanteus* | EANAH604-12 | ROM Herps 13494 | KU986044 |
| *Crotalus adamanteus* | EANAH736-12 | ROM Herps 18130 | KU985639 |
| *Crotalus adamanteus* | EANAH737-12 | ROM Herps 18131 | KU986222 |
| *Crotalus atrox* | EANAH725-12 | ROM Herps 15279 | KU986265 |
| *Crotalus atrox* | EANAH743-12 | ROM Herps 18144 | KU986260 |
| *Crotalus atrox* | EANAH744-12 | ROM Herps 18149 | KU985911 |
| *Crotalus atrox* | EANAH836-12 | ROM Herps 23298 | KU985749 |
| *Crotalus atrox* | EANAH837-12 | ROM Herps 23299 | KU986161 |
| *Crotalus cerastes* | EANAH420-12 | BIOUG-PW002 | KU985577 |
| *Crotalus cerastes* | EANAH642-12 | ROM Herps 13820 | KU986030 |
| *Crotalus cerastes* | EANAH643-12 | ROM Herps 13821 | KU985599 |
| *Crotalus cerastes* | EANAH772-12 | ROM Herps 19752 | KU986105 |
| *Crotalus cerastes* | EANAH789-12 | ROM Herps 21102 | KU985812 |
| *Crotalus cerastes* | EANAH790-12 | ROM Herps 21115 | KU986225 |
| *Crotalus horridus* | EANAH738-12 | ROM Herps 18132 | KU986206 |
| *Crotalus horridus* | EANAH739-12 | ROM Herps 18133 | KU986223 |
| *Crotalus lepidus* | EANAH445-12 | BIOUG-PW027 | KU985870 |
| *Crotalus lepidus* | EANAH735-12 | ROM Herps 18127 | KU985625 |
| *Crotalus lepidus* | EANAH871-12 | ROM Herps 42403 | KU985695 |
| *Crotalus lepidus* | EANAH872-12 | ROM Herps 42415 | KU985715 |
| *Crotalus lepidus klauberi* | EANAH426-12 | BIOUG-PW008 | KU985897 |
| *Crotalus mitchellii* | EANAH751-12 | ROM Herps 18176 | KU986000 |
| *Crotalus mitchellii* | EANAH752-12 | ROM Herps 18177 | KU986076 |
| *Crotalus mitchellii* | EANAH753-12 | ROM Herps 18178 | KU985590 |
| *Crotalus mitchellii* | EANAH754-12 | ROM Herps 18179 | KU985982 |
| *Crotalus mitchellii* | EANAH755-12 | ROM Herps 18181 | KU985631 |
| *Crotalus mitchellii* | EANAH909-12 | SDNHM Field 3125 | KU985616 |
| *Crotalus molossus* | EANAH724-12 | ROM Herps 15277 | KU986060 |
| *Crotalus molossus* | EANAH726-12 | ROM Herps 15287 | KU986122 |
| *Crotalus molossus* | EANAH740-12 | ROM Herps 18140 | KU985804 |
| *Crotalus molossus* | EANAH741-12 | ROM Herps 18141 | KU985681 |
| *Crotalus molossus* | EANAH742-12 | ROM Herps 18142 | KU985682 |
| *Crotalus oreganus* | EANAH904-12 | SDNHM Field 3112 | KU985566 |
| *Crotalus pricei* | EANAH1000-12 | ROM Herps 47095b | KU986213 |
| *Crotalus pricei* | EANAH1095-13 | ROM Herps 45252a | KU985896 |
| *Crotalus pricei* | EANAH745-12 | ROM Herps 18157 | KU985692 |
| *Crotalus pricei* | EANAH746-12 | ROM Herps 18159 | KU985898 |
| *Crotalus pricei* | EANAH897-12 | ROM Herps 45256 | KU986103 |
| *Crotalus pricei* | EANAH903-12 | ROM Herps 47095 | KU986275 |
| *Crotalus pricei* | EANAH999-12 | ROM Herps 45256b | KU985934 |
| *Crotalus ruber* | EANAH1090-13 | ROM Herps 18198a | KU985805 |
| *Crotalus ruber* | EANAH760-12 | ROM Herps 18203 | KU985662 |
| *Crotalus ruber* | EANAH761-12 | ROM Herps 18204 | KU986278 |
| *Crotalus ruber* | EANAH762-12 | ROM Herps 18205 | KU985814 |
| *Crotalus ruber* | EANAH763-12 | ROM Herps 18207 | KU985635 |
| *Crotalus ruber* | EANAH908-12 | SDNHM Field 3124 | KU985987 |
| *Crotalus scutulatus* | EANAH602-12 | ROM Herps 13486 | KU985687 |
| *Crotalus scutulatus* | EANAH764-12 | ROM Herps 18208 | KU985676 |
| *Crotalus scutulatus* | EANAH765-12 | ROM Herps 18210 | KU986166 |
| *Crotalus scutulatus* | EANAH766-12 | ROM Herps 18223 | KU985747 |
| *Crotalus tigris* | EANAH1089-13 | ROM Herps 19773a | KU986057 |
| *Crotalus tigris* | EANAH747-12 | ROM Herps 18167 | KU985970 |
| *Crotalus tigris* | EANAH748-12 | ROM Herps 18168 | KU985565 |
| *Crotalus tigris* | EANAH749-12 | ROM Herps 18169 | KU985878 |
| *Crotalus tigris* | EANAH750-12 | ROM Herps 18171 | KU985821 |
| *Crotalus viridis* | EANAH774-12 | ROM Herps 19757 | KU985787 |
| *Crotalus viridis* | EANAH775-12 | ROM Herps 19761 | KU985701 |
| *Crotalus viridis* | EANAH776-12 | ROM Herps 19764 | KU985831 |
| *Crotalus willardi* | EANAH756-12 | ROM Herps 18183 | KU985952 |
| *Crotalus willardi* | EANAH757-12 | ROM Herps 18184 | KU986183 |
| *Crotalus willardi* | EANAH758-12 | ROM Herps 18185 | KU986075 |
| *Crotaphytus bicinctores* | EANAH696-12 | ROM Herps 14579 | KU986221 |
| *Crotaphytus bicinctores* | EANAH697-12 | ROM Herps 14580 | KU985832 |
| *Crotaphytus bicinctores* | EANAH698-12 | ROM Herps 14581 | KU986114 |
| *Crotaphytus bicinctores* | EANAH700-12 | ROM Herps 14584 | KU985819 |
| *Crotaphytus bicinctores* | EANAH705-12 | ROM Herps 14748 | KU986003 |
| *Crotaphytus collaris* | EANAH716-12 | ROM Herps 15051 | KU985978 |
| *Crotaphytus nebrius* | EANAH948-12 | MVZ Herp 244244 | KU985558 |
| *Crotaphytus vestigium* | EANAH608-12 | ROM Herps 13557 | KU986034 |
| *Crotaphytus vestigium* | EANAH609-12 | ROM Herps 13558 | KU985671 |
| *Crotaphytus vestigium* | EANAH610-12 | ROM Herps 13573 | KU985891 |
| *Crotaphytus vestigium* | EANAH611-12 | ROM Herps 13574 | KU985557 |
| *Deirochelys reticularia* | EANAH945-12 | MVZ Herp 137744 | KU985791 |
| *Dipsosaurus dorsalis* | EANAH605-12 | ROM Herps 13534 | KU986229 |
| *Dipsosaurus dorsalis* | EANAH607-12 | ROM Herps 13556 | KU986181 |
| *Dipsosaurus dorsalis* | EANAH637-12 | ROM Herps 13785 | KU985572 |
| *Dipsosaurus dorsalis* | EANAH645-12 | ROM Herps 13842 | KU986065 |
| *Dipsosaurus dorsalis* | EANAH650-12 | ROM Herps 13891 | KU986120 |
| *Elgaria panamintina* | EANAH931-12 | MVZ Herp 191076 | KU985779 |
| *Emys blandingii* | EANAH728-12 | ROM Herps 15872 | KU986250 |
| *Emys blandingii* | EANAH729-12 | ROM Herps 15873 | KU985739 |
| *Emys blandingii* | EANAH730-12 | ROM Herps 15874 | KU985909 |
| *Emys blandingii* | EANAH783-12 | ROM Herps 20922 | KU985825 |
| *Emys blandingii* | EANAH784-12 | ROM Herps 20923 | KU985853 |
| *Emys marmorata* | EANAH946-12 | MVZ Herp 137811 | KU986112 |
| *Farancia abacura* | EANAH473-12 | FMNH Herps 257099 | KU985610 |
| *Farancia abacura* | EANAH487-12 | FMNH Herps 257860 | KU986009 |
| *Farancia abacura* | EANAH551-12 | KU KUH 289533 | KU985744 |
| *Gambelia copei* | EANAH949-12 | MVZ Herp 161174 | KU986182 |
| *Gambelia wislizenii* | EANAH1091-13 | ROM Herps 14021a | KU986224 |
| *Gambelia wislizenii* | EANAH634-12 | ROM Herps 13775 | KU985864 |
| *Gambelia wislizenii* | EANAH647-12 | ROM Herps 13852 | KU986121 |
| *Gambelia wislizenii* | EANAH660-12 | ROM Herps 13987 | KU985877 |
| *Gambelia wislizenii* | EANAH661-12 | ROM Herps 13988 | KU985618 |
| *Graptemys geographica* | EANAH785-12 | ROM Herps 20931 | KU985724 |
| *Gyalopion canum* | EANAH934-12 | MVZ Herp 231854 | KU985598 |
| *Heloderma horridum* | EANAH900-12 | ROM Herps 46492 | KU986270 |
| *Hemidactylus turcicus* | EANAH925-12 | UAHC Main 15554 | KU986144 |
| *Heterodon nasicus* | EANAH409-12 | AMNH Herpetology 139228 | KU985658 |
| *Heterodon nasicus* | EANAH469-12 | FMNH Herps 251144 | KU985763 |
| *Heterodon platirhinos* | EANAH471-12 | FMNH Herps 257022 | KU986007 |
| *Heterodon platirhinos* | EANAH543-12 | FMNH Herps 282473 | KU985930 |
| *Heterodon platirhinos* | EANAH859-12 | ROM Herps 26217 | KU986023 |
| *Holbrookia maculata* | EANAH711-12 | ROM Herps 14960 | KU985985 |
| *Holbrookia maculata* | EANAH712-12 | ROM Herps 14963 | KU986059 |
| *Holbrookia maculata* | EANAH714-12 | ROM Herps 14966 | KU985954 |
| *Holbrookia maculata* | EANAH718-12 | ROM Herps 15095 | KU985569 |
| *Holbrookia maculata* | EANAH719-12 | ROM Herps 15096 | KU985690 |
| *Hypsiglena chlorophaea* | EANAH466-12 | BYUH Main 42373 | KU986232 |
| *Hypsiglena chlorophaea* | EANAH467-12 | BYUH Main 42376 | KU985654 |
| *Hypsiglena chlorophaea* | EANAH468-12 | BYUH Main 42832 | KU985727 |
| *Hypsiglena ochrorhyncha* | EANAH693-12 | ROM Herps 14478 | KU985731 |
| *Lampropeltis calligaster* | EANAH546-12 | KU KUH 289508 | KU985581 |
| *Lampropeltis calligaster* | EANAH548-12 | KU KUH 289517 | KU986150 |
| *Lampropeltis calligaster* | EANAH552-12 | KU KUH 289534 | KU985921 |
| *Lampropeltis getula* | EANAH1098-13 | ROM Herps 13721a | KU985672 |
| *Lampropeltis getula* | EANAH617-12 | ROM Herps 13666 | KU985981 |
| *Lampropeltis getula* | EANAH619-12 | ROM Herps 13688 | KU985960 |
| *Lampropeltis getula* | EANAH624-12 | ROM Herps 13720 | KU986273 |
| *Lampropeltis getula* | EANAH626-12 | ROM Herps 13727 | KU986082 |
| *Lampropeltis triangulum* | EANAH417-12 | BIOUG-EAC001 | KU986088 |
| *Lampropeltis triangulum* | EANAH508-12 | FMNH Herps 266870 | KU985601 |
| *Lampropeltis triangulum* | EANAH515-12 | FMNH Herps 268700 | KU985694 |
| *Lampropeltis zonata* | EANAH843-12 | ROM Herps 23327 | KU986141 |
| *Leptodeira septentrionalis* | EANAH544-12 | KU KUH 218419 | KU985849 |
| *Leptodeira septentrionalis* | EANAH576-12 | KU KUH 291311 | KU986063 |
| *Leptodeira septentrionalis* | EANAH577-12 | KU KUH 291316 | KU985773 |
| *Leptotyphlops dulcis* | EANAH954-12 | MVZ Herp 230602 | KU985576 |
| *Leptotyphlops humilis* | EANAH594-12 | MVZ Herp 161569 | KU986194 |
| *Leptotyphlops humilis* | EANAH895-12 | ROM Herps 43773 | KU985852 |
| *Leptotyphlops humilis* | EANAH898-12 | ROM Herps 45259 | KU985605 |
| *Lichanura trivirgata* | EANAH910-12 | SDNHM Field 3126 | KU985951 |
| *Masticophis bilineatus* | EANAH407-12 | AMNH Herpetology 138215 | KU985586 |
| *Masticophis bilineatus* | EANAH713-12 | ROM Herps 14965 | KU986198 |
| *Masticophis flagellum* | EANAH704-12 | ROM Herps 14716 | KU985718 |
| *Masticophis flagellum* | EANAH792-12 | ROM Herps 22648 | KU986136 |
| *Masticophis flagellum* | EANAH793-12 | ROM Herps 22649 | KU986192 |
| *Masticophis flagellum* | EANAH794-12 | ROM Herps 22650 | KU986178 |
| *Masticophis flagellum* | EANAH798-12 | ROM Herps 22722 | KU985931 |
| *Masticophis lateralis* | EANAH935-12 | MVZ Herp 229146 | KU986267 |
| *Masticophis schotti* | EANAH936-12 | MVZ Herp 233302 | KU985965 |
| *Masticophis taeniatus* | EANAH960-12 | MVZ Herp 235923 | KU986140 |
| *Nerodia cyclopion* | EANAH916-12 | UAHC Main 15198 | KU985643 |
| *Nerodia cyclopion* | EANAH917-12 | UAHC Main 15202 | KU985811 |
| *Nerodia cyclopion* | EANAH918-12 | UAHC Main 15203 | KU985822 |
| *Nerodia cyclopion* | EANAH919-12 | UAHC Main 15204 | KU986186 |
| *Nerodia erythrogaster* | EANAH481-12 | FMNH Herps 257454 | KU986256 |
| *Nerodia erythrogaster* | EANAH513-12 | FMNH Herps 268687 | KU985644 |
| *Nerodia erythrogaster* | EANAH514-12 | FMNH Herps 268688 | KU985950 |
| *Nerodia fasciata* | EANAH556-12 | KU KUH 289567 | KU985722 |
| *Nerodia rhombifer* | EANAH480-12 | FMNH Herps 257453 | KU986145 |
| *Nerodia rhombifer* | EANAH567-12 | KU KUH 290677 | KU985924 |
| *Nerodia rhombifer* | EANAH568-12 | KU KUH 290678 | KU986160 |
| *Nerodia rhombifer* | EANAH573-12 | KU KUH 290690 | KU986220 |
| *Nerodia sipedon* | EANAH491-12 | FMNH Herps 265167 | KU985915 |
| *Nerodia sipedon* | EANAH529-12 | FMNH Herps 274107 | KU985556 |
| *Nerodia taxispilota* | EANAH920-12 | UAHC Main 15229 | KU986012 |
| *Nerodia taxispilota* | EANAH921-12 | UAHC Main 15234 | KU985670 |
| *Opheodrys aestivus* | EANAH915-12 | UAHC Main 14598 | KU985612 |
| *Opheodrys aestivus* | EANAH926-12 | UAHC Main 15557 | KU985594 |
| *Opheodrys aestivus* | EANAH927-12 | UAHC Main 15562 | KU985808 |
| *Opheodrys vernalis* | EANAH478-12 | FMNH Herps 257449 | KU986002 |
| *Opheodrys vernalis* | EANAH490-12 | FMNH Herps 262220 | KU985827 |
| *Opheodrys vernalis* | EANAH512-12 | FMNH Herps 267679 | KU985824 |
| *Ophisaurus attenuatus* | EANAH510-12 | FMNH Herps 267547 | KU985933 |
| *Ophisaurus attenuatus* | EANAH539-12 | FMNH Herps 275647 | KU985962 |
| *Ophisaurus attenuatus* | EANAH540-12 | FMNH Herps 277121 | KU985790 |
| *Ophisaurus attenuatus* | EANAH583-12 | KU KUH 307850 | KU985620 |
| *Ophisaurus attenuatus* | EANAH584-12 | KU KUH 307851 | KU985998 |
| *Ophisaurus attenuatus* | EANAH586-12 | KU KUH 307860 | KU985760 |
| *Ophisaurus compressus* | EANAH501-12 | FMNH Herps 266588 | KU985652 |
| *Ophisaurus ventralis* | EANAH489-12 | FMNH Herps 262217 | KU986014 |
| *Ophisaurus ventralis* | EANAH502-12 | FMNH Herps 266589 | KU985615 |
| *Oxybelis aeneus* | EANAH564-12 | KU KUH 289907 | KU985830 |
| *Oxybelis aeneus* | EANAH860-12 | ROM Herps 28374 | KU985609 |
| *Pantherophis guttatus* | EANAH418-12 | BIOUG-EAC002 | KU986233 |
| *Pantherophis guttatus* | EANAH499-12 | FMNH Herps 266586 | KU985957 |
| *Pantherophis guttatus* | EANAH503-12 | FMNH Herps 266590 | KU985583 |
| *Pantherophis guttatus* | EANAH923-12 | UAHC Main 15551 | KU986054 |
| *Pantherophis guttatus* | EANAH924-12 | UAHC Main 15552 | KU985996 |
| *Pantherophis guttatus* | EANAH928-12 | UAHC Main 15588 | KU986236 |
| *Pantherophis obsoletus* | EANAH542-12 | FMNH Herps 281640 | KU985961 |
| *Pantherophis obsoletus* | EANAH791-12 | ROM Herps 21937 | KU986043 |
| *Pantherophis obsoletus* | EANAH854-12 | ROM Herps 24661 | KU985879 |
| *Pantherophis obsoletus* | EANAH855-12 | ROM Herps 24662 | KU985858 |
| *Pantherophis obsoletus* | EANAH856-12 | ROM Herps 24663 | KU985940 |
| *Pantherophis obsoletus* | EANAH857-12 | ROM Herps 24664 | KU985993 |
| *Pantherophis vulpinus* | EANAH486-12 | FMNH Herps 257481 | KU985963 |
| *Pantherophis vulpinus* | EANAH492-12 | FMNH Herps 265169 | KU986073 |
| *Pantherophis vulpinus* | EANAH497-12 | FMNH Herps 266522 | KU986157 |
| *Petrosaurus mearnsi* | EANAH648-12 | ROM Herps 13859 | KU985980 |
| *Petrosaurus mearnsi* | EANAH662-12 | ROM Herps 14015 | KU986135 |
| *Petrosaurus mearnsi* | EANAH663-12 | ROM Herps 14016 | KU986156 |
| *Petrosaurus mearnsi* | EANAH664-12 | ROM Herps 14017 | KU985622 |
| *Petrosaurus mearnsi* | EANAH665-12 | ROM Herps 14018 | KU985580 |
| *Phrynosoma cornutum* | EANAH398-12 | AMNH Herpetology 137691 | KU986276 |
| *Phrynosoma cornutum* | EANAH399-12 | AMNH Herpetology 137692 | KU986066 |
| *Phrynosoma hernandesi* | EANAH950-12 | MVZ Herp 245875 | KU985667 |
| *Phrynosoma mcallii* | EANAH651-12 | ROM Herps 13899 | KU986020 |
| *Phrynosoma mcallii* | EANAH668-12 | ROM Herps 14026 | KU985720 |
| *Phrynosoma modestum* | EANAH400-12 | AMNH Herpetology 137693 | KU986259 |
| *Phrynosoma modestum* | EANAH401-12 | AMNH Herpetology 137694 | KU985646 |
| *Phyllorhynchus browni* | EANAH707-12 | ROM Herps 14898 | KU985885 |
| *Phyllorhynchus browni* | EANAH708-12 | ROM Herps 14899 | KU986242 |
| *Phyllorhynchus decurtatus* | EANAH1092-13 | ROM Herps 14151a | KU985573 |
| *Phyllorhynchus decurtatus* | EANAH678-12 | ROM Herps 14152 | KU985989 |
| *Phyllorhynchus decurtatus* | EANAH679-12 | ROM Herps 14153 | KU985942 |
| *Pituophis catenifer* | EANAH470-12 | FMNH Herps 251260 | KU986152 |
| *Pituophis catenifer* | EANAH531-12 | FMNH Herps 274776 | KU985571 |
| *Pituophis catenifer* | EANAH534-12 | FMNH Herps 274803 | KU985847 |
| *Pituophis catenifer* | EANAH912-12 | SDNHM Field 3134 | KU985810 |
| *Pituophis melanoleucus* | EANAH451-12 | BIOUG-PW033 | KU985579 |
| *Pituophis melanoleucus* | EANAH622-12 | ROM Herps 13697 | KU985562 |
| *Pituophis melanoleucus* | EANAH680-12 | ROM Herps 14158 | KU986219 |
| *Pituophis melanoleucus* | EANAH681-12 | ROM Herps 14159 | KU986262 |
| *Pituophis melanoleucus* | EANAH682-12 | ROM Herps 14160 | KU986018 |
| *Pituophis melanoleucus* | EANAH844-12 | ROM Herps 23329 | KU986266 |
| *Plestiodon anthracinus* | EANAH894-12 | ROM Herps 43736 | KU985925 |
| *Plestiodon fasciatus* | EANAH570-12 | KU KUH 290684 | KU985793 |
| *Plestiodon fasciatus* | EANAH571-12 | KU KUH 290686 | KU986226 |
| *Plestiodon fasciatus* | EANAH585-12 | KU KUH 307857 | KU985881 |
| *Plestiodon gilberti* | EANAH812-12 | ROM Herps 23239 | KU985907 |
| *Plestiodon gilberti* | EANAH813-12 | ROM Herps 23240 | KU985603 |
| *Plestiodon gilberti* | EANAH814-12 | ROM Herps 23241 | KU986032 |
| *Plestiodon gilberti* | EANAH815-12 | ROM Herps 23242 | KU986068 |
| *Plestiodon inexpectatus* | EANAH822-12 | ROM Herps 23249 | KU985659 |
| *Plestiodon laticeps* | EANAA077-12 | FMNH Herps 259981 | KU985588 |
| *Plestiodon obsoletus* | EANAH955-12 | MVZ Herp 137633 | KU986246 |
| *Plestiodon skiltonianus* | EANAA002-12 | SDNHM Field 3108 | KU985613 |
| *Plestiodon skiltonianus* | EANAH817-12 | ROM Herps 23244 | KU986048 |
| *Plestiodon skiltonianus* | EANAH818-12 | ROM Herps 23245 | KU986179 |
| *Plestiodon skiltonianus* | EANAH819-12 | ROM Herps 23246 | KU985914 |
| *Plestiodon skiltonianus* | EANAH820-12 | ROM Herps 23247 | KU985937 |
| *Plestiodon skiltonianus* | EANAH821-12 | ROM Herps 23248 | KU985992 |
| *Regina grahamii* | EANAH538-12 | FMNH Herps 275641 | KU985717 |
| *Regina septemvittata* | EANAH484-12 | FMNH Herps 257458 | KU985947 |
| *Regina septemvittata* | EANAH528-12 | FMNH Herps 274027 | KU985899 |
| *Regina septemvittata* | EANAH530-12 | FMNH Herps 274746 | KU986238 |
| *Rhadinaea flavilata* | EANAH938-12 | MVZ Herp 164867 | KU986139 |
| *Rhinocheilus lecontei* | EANAH690-12 | ROM Herps 14456 | KU986197 |
| *Rhinocheilus lecontei* | EANAH694-12 | ROM Herps 14480 | KU985733 |
| *Rhinocheilus lecontei* | EANAH706-12 | ROM Herps 14896 | KU985624 |
| *Rhinocheilus lecontei* | EANAH710-12 | ROM Herps 14958 | KU985883 |
| *Rhinocheilus lecontei* | EANAH721-12 | ROM Herps 15265 | KU985737 |
| *Salvadora grahamiae* | EANAH410-12 | AMNH Herpetology 142217 | KU986187 |
| *Salvadora grahamiae* | EANAH842-12 | ROM Herps 23326 | KU985949 |
| *Salvadora hexalepis* | EANAH411-12 | AMNH Herpetology 143041 | KU985946 |
| *Salvadora hexalepis* | EANAH415-12 | AMNH Herpetology 146740 | KU985752 |
| *Salvadora hexalepis* | EANAH606-12 | ROM Herps 13548 | KU985871 |
| *Salvadora hexalepis* | EANAH773-12 | ROM Herps 19755 | KU985862 |
| *Salvadora hexalepis* | EANAH841-12 | ROM Herps 23325 | KU985856 |
| *Sauromalus ater* | EANAH588-12 | MVZ Herp 161257 | KU986079 |
| *Sauromalus ater* | EANAH613-12 | ROM Herps 13637 | KU985991 |
| *Sauromalus ater* | EANAH614-12 | ROM Herps 13638 | KU986149 |
| *Sauromalus ater* | EANAH809-12 | ROM Herps 23224 | KU985584 |
| *Sauromalus ater* | EANAH832-12 | ROM Herps 23286 | KU985705 |
| *Sauromalus ater* | EANAH911-12 | SDNHM Field 3127 | KU986041 |
| *Sceloporus arenicolus* | EANAH952-12 | MVZ Herp 230567 | KU986046 |
| *Sceloporus graciosus* | EANAH823-12 | ROM Herps 23250 | KU986093 |
| *Sceloporus graciosus* | EANAH824-12 | ROM Herps 23259 | KU985796 |
| *Sceloporus graciosus* | EANAH827-12 | ROM Herps 23262 | KU985781 |
| *Sceloporus graciosus* | EANAH828-12 | ROM Herps 23263 | KU986218 |
| *Sceloporus graciosus* | EANAH858-12 | ROM Herps 26193 | KU985673 |
| *Sceloporus jarrovii* | EANAH404-12 | AMNH Herpetology 137701 | KU986031 |
| *Sceloporus jarrovii* | EANAH405-12 | AMNH Herpetology 137702 | KU985955 |
| *Sceloporus magister* | EANAH630-12 | ROM Herps 13758 | KU986167 |
| *Sceloporus magister* | EANAH702-12 | ROM Herps 14610 | KU985969 |
| *Sceloporus magister* | EANAH833-12 | ROM Herps 23289 | KU985974 |
| *Sceloporus magister* | EANAH834-12 | ROM Herps 23290 | KU985765 |
| *Sceloporus occidentalis* | EANAH906-12 | SDNHM Field 3118 | KU986255 |
| *Sceloporus occidentalis* | EANAH996-12 | SDNHM Field 3118b | KU986109 |
| *Sceloporus orcutti* | EANAH683-12 | ROM Herps 14402 | KU986248 |
| *Sceloporus orcutti* | EANAH689-12 | ROM Herps 14453 | KU985935 |
| *Sceloporus orcutti* | EANAH703-12 | ROM Herps 14673 | KU985842 |
| *Sceloporus orcutti* | EANAH810-12 | ROM Herps 23226 | KU986155 |
| *Sceloporus orcutti* | EANAH811-12 | ROM Herps 23238 | KU986164 |
| *Sceloporus orcutti* | EANAH905-12 | SDNHM Field 3114 | KU986053 |
| *Sceloporus undulatus* | EANAH825-12 | ROM Herps 23260 | KU986028 |
| *Sceloporus undulatus* | EANAH826-12 | ROM Herps 23261 | KU985799 |
| *Sceloporus undulatus* | EANAH829-12 | ROM Herps 23264 | KU985640 |
| *Sceloporus undulatus* | EANAH830-12 | ROM Herps 23265 | KU986134 |
| *Sceloporus undulatus* | EANAH831-12 | ROM Herps 23266 | KU985686 |
| *Sceloporus variabilis* | EANAH565-12 | KU KUH 290014 | KU986208 |
| *Sceloporus variabilis* | EANAH575-12 | KU KUH 291284 | KU985786 |
| *Sceloporus variabilis* | EANAH578-12 | KU KUH 291342 | KU985956 |
| *Sceloporus virgatus* | EANAH1096-13 | ROM Herps 15269a | KU985702 |
| *Sceloporus virgatus* | EANAH402-12 | AMNH Herpetology 137699 | KU985908 |
| *Sceloporus virgatus* | EANAH403-12 | AMNH Herpetology 137700 | KU985944 |
| *Scincella lateralis* | EANAH572-12 | KU KUH 290688 | KU986201 |
| *Scincella lateralis* | EANAH892-12 | ROM Herps 43725 | KU986230 |
| *Scincella lateralis* | EANAH893-12 | ROM Herps 43728 | KU985918 |
| *Seminatrix pygaea* | EANAH488-12 | FMNH Herps 257861 | KU986095 |
| *Senticolis triaspis* | EANAH408-12 | AMNH Herpetology 138485 | KU985888 |
| *Sistrurus catenatus* | EANAH852-12 | ROM Herps 24592 | KU985958 |
| *Sistrurus catenatus* | EANAH853-12 | ROM Herps 24593 | KU985651 |
| *Sistrurus catenatus* | EANAH863-12 | ROM Herps 41217 | KU986172 |
| *Sistrurus catenatus* | EANAH864-12 | ROM Herps 41218 | KU986216 |
| *Sistrurus miliarius* | EANAH603-12 | ROM Herps 13493 | KU985759 |
| *Sistrurus miliarius* | EANAH768-12 | ROM Herps 18232 | KU985807 |
| *Sistrurus miliarius* | EANAH769-12 | ROM Herps 18233 | KU986235 |
| *Sistrurus miliarius* | EANAH770-12 | ROM Herps 18234 | KU985845 |
| *Sistrurus miliarius* | EANAH771-12 | ROM Herps 18235 | KU986085 |
| *Sonora semiannulata* | EANAH939-12 | MVZ Herp 164965 | KU986151 |
| *Sternotherus carinatus* | EANAH953-12 | MVZ Herp 238127 | KU986153 |
| *Sternotherus odoratus* | EANAH507-12 | FMNH Herps 266862 | KU986193 |
| *Sternotherus odoratus* | EANAH545-12 | KU KUH 289503 | KU985756 |
| *Storeria dekayi* | EANAH532-12 | FMNH Herps 274790 | KU985887 |
| *Storeria dekayi* | EANAH535-12 | FMNH Herps 274807 | KU986037 |
| *Storeria dekayi* | EANAH557-12 | KU KUH 289589 | KU986092 |
| *Storeria dekayi* | EANAH561-12 | KU KUH 289633 | KU986264 |
| *Storeria dekayi* | EANAH579-12 | KU KUH 307801 | KU985755 |
| *Storeria occipitomaculata* | EANAH477-12 | FMNH Herps 257448 | KU985725 |
| *Storeria occipitomaculata* | EANAH498-12 | FMNH Herps 266535 | KU985854 |
| *Storeria occipitomaculata* | EANAH526-12 | FMNH Herps 271422 | KU986005 |
| *Storeria occipitomaculata* | EANAH553-12 | KU KUH 289547 | KU986214 |
| *Storeria occipitomaculata* | EANAH554-12 | KU KUH 289548 | KU986015 |
| *Tantilla hobartsmithi* | EANAH940-12 | MVZ Herp 225756 | KU986035 |
| *Tantilla nigriceps* | EANAH941-12 | MVZ Herp 225757 | KU986195 |
| *Tantilla relicta* | EANAH942-12 | MVZ Herp 164969 | KU985627 |
| *Tantilla yaquia* | EANAH397-12 | AMNH Herpetology 136146 | KU985666 |
| *Terrapene carolina* | EANAH786-12 | ROM Herps 20933 | KU985568 |
| *Thamnophis brachystoma* | EANAH943-12 | MVZ Herp 137702 | KU986124 |
| *Thamnophis butleri* | EANAH479-12 | FMNH Herps 257451 | KU985778 |
| *Thamnophis couchii* | EANAH517-12 | FMNH Herps 268719 | KU985604 |
| *Thamnophis cyrtopsis* | EANAH406-12 | AMNH Herpetology 138214 | KU985665 |
| *Thamnophis cyrtopsis* | EANAH474-12 | FMNH Herps 257124 | KU985745 |
| *Thamnophis marcianus* | EANAH944-12 | MVZ Herp 244073 | KU985591 |
| *Thamnophis ordinoides* | EANAH839-12 | ROM Herps 23321 | KU985997 |
| *Thamnophis proximus* | EANAH482-12 | FMNH Herps 257456 | KU985653 |
| *Thamnophis proximus* | EANAH516-12 | FMNH Herps 268706 | KU985895 |
| *Thamnophis proximus* | EANAH527-12 | FMNH Herps 271424 | KU986029 |
| *Thamnophis radix* | EANAH476-12 | FMNH Herps 257447 | KU985714 |
| *Thamnophis radix* | EANAH493-12 | FMNH Herps 265277 | KU986268 |
| *Thamnophis radix* | EANAH840-12 | ROM Herps 23322 | KU985973 |
| *Thamnophis sauritus* | EANAH494-12 | FMNH Herps 265280 | KU985906 |
| *Thamnophis sauritus* | EANAH533-12 | FMNH Herps 274798 | KU985691 |
| *Thamnophis sauritus* | EANAH541-12 | FMNH Herps 281632 | KU986143 |
| *Trachemys scripta* | EANAH485-12 | FMNH Herps 257462 | KU985945 |
| *Trachemys scripta* | EANAH511-12 | FMNH Herps 267587 | KU986207 |
| *Trachemys scripta* | EANAH800-12 | ROM Herps 22882 | KU985679 |
| *Typhlops vermicularis* | EANAH849-12 | ROM Herps 23403 | KU985938 |
| *Uma notata* | EANAH653-12 | ROM Herps 13927 | KU986013 |
| *Uma notata* | EANAH654-12 | ROM Herps 13928 | KU986185 |
| *Uma notata* | EANAH655-12 | ROM Herps 13929 | KU985674 |
| *Uma notata* | EANAH656-12 | ROM Herps 13930 | KU986199 |
| *Uma notata* | EANAH657-12 | ROM Herps 13949 | KU985990 |
| *Uma notata cowlesi* | EANAH993-12 | MCZ Herp R-62415 | KU985657 |
| *Urosaurus graciosus* | EANAH646-12 | ROM Herps 13844 | KU985873 |
| *Urosaurus graciosus* | EANAH652-12 | ROM Herps 13900 | KU985684 |
| *Urosaurus graciosus* | EANAH881-12 | ROM Herps 43494 | KU986245 |
| *Urosaurus graciosus* | EANAH882-12 | ROM Herps 43495 | KU985693 |
| *Urosaurus graciosus* | EANAH883-12 | ROM Herps 43496 | KU985785 |
| *Urosaurus nigricaudus* | EANAH589-12 | MVZ Herp 161339 | KU986083 |
| *Urosaurus nigricaudus* | EANAH590-12 | MVZ Herp 161340 | KU986118 |
| *Urosaurus nigricaudus* | EANAH591-12 | MVZ Herp 161341 | KU985894 |
| *Urosaurus nigricaudus* | EANAH592-12 | MVZ Herp 161342 | KU985698 |
| *Urosaurus nigricaudus* | EANAH593-12 | MVZ Herp 161343 | KU985823 |
| *Urosaurus ornatus* | EANAH877-12 | ROM Herps 43482 | KU986004 |
| *Urosaurus ornatus* | EANAH878-12 | ROM Herps 43483 | KU986133 |
| *Urosaurus ornatus* | EANAH879-12 | ROM Herps 43484 | KU985789 |
| *Urosaurus ornatus* | EANAH880-12 | ROM Herps 43485 | KU986067 |
| *Urosaurus ornatus* | EANAH914-12 | TCWC Herps 58664 | KU985893 |
| *Uta stansburiana* | EANAH673-12 | ROM Herps 14044 | KU986039 |
| *Uta stansburiana* | EANAH699-12 | ROM Herps 14582 | KU985865 |
| *Uta stansburiana* | EANAH701-12 | ROM Herps 14587 | KU986052 |
| *Uta stansburiana* | EANAH901-12 | ROM Herps 46884 | KU985753 |
| *Uta stansburiana* | EANAH902-12 | ROM Herps 46885 | KU986271 |
| *Uta stansburiana* | EANAH913-12 | SDNHM Field 3136 | KU986071 |
| *Virginia valeriae* | EANAH547-12 | KU KUH 289510 | KU986252 |
| *Virginia valeriae* | EANAH550-12 | KU KUH 289529 | KU985953 |
| *Xantusia arizonae* | EANAH957-12 | MVZ Herp 232602 | KU985939 |
| *Xantusia bezyi* | EANAH958-12 | MVZ Herp 232606 | KU985801 |
| *Xantusia henshawi* | EANAH684-12 | ROM Herps 14407 | KU986129 |
| *Xantusia henshawi* | EANAH685-12 | ROM Herps 14408 | KU985916 |
| *Xantusia henshawi* | EANAH686-12 | ROM Herps 14409 | KU985994 |
| *Xantusia henshawi* | EANAH687-12 | ROM Herps 14410 | KU985797 |
| *Xantusia henshawi* | EANAH688-12 | ROM Herps 14411 | KU986104 |
| *Xantusia henshawi* | EANAH907-12 | SDNHM Field 3123 | KU986217 |
| *Xantusia vigilis* | EANAH631-12 | ROM Herps 13772 | KU985802 |
| *Xantusia vigilis* | EANAH632-12 | ROM Herps 13773 | KU986173 |
| *Xantusia vigilis* | EANAH633-12 | ROM Herps 13774 | KU985928 |
| *Xantusia vigilis* | EANAH658-12 | ROM Herps 13964 | KU985829 |
| *Xantusia vigilis* | EANAH659-12 | ROM Herps 13967 | KU986189 |
| *Xantusia wigginsi* | EANAH959-12 | MVZ Herp 236378 | KU985848 |
| **Non-North American reptiles** |  |  |  |
| *Agama agama* | EANAH780-12 | ROM Herps 19897 | KU986331 |
| *Agkistrodon ussuriensis* | EANAH781-12 | ROM Herps 20455 | KU986337 |
| *Anolis extremus* | EANAH782-12 | ROM Herps 20571 | KU986314 |
| *Bitis arietans* | EANAH424-12 | BIOUG-PW006 | KU986340 |
| *Boa constrictor* | EANAH452-12 | BIOUG-PW034 | KU986329 |
| *Boiga dendrophila* | EANAH455-12 | BIOUG-PW037 | KU986332 |
| *Boiga irregularis* | EANAH845-12 | ROM Herps 23335 | KU986313 |
| *Boiga* sp. | EANAH454-12 | BIOUG-PW036 | KU986322 |
| *Bothriechis lateralis* | EANAH429-12 | BIOUG-PW011 | KU986325 |
| *Bothriechis lateralis* | EANAH430-12 | BIOUG-PW012 | KU986323 |
| *Bothriechis lateralis* | EANAH450-12 | BIOUG-PW032 | KU986328 |
| *Bothriechis schlegelii* | EANAH419-12 | BIOUG-PW001 | KU986324 |
| *Bothrops asper* | EANAH434-12 | BIOUG-PW016 | KU986307 |
| *Bothrops atrox* | EANAH799-12 | ROM Herps 22848 | KU986342 |
| *Bothrops barnetti* | EANAH423-12 | BIOUG-PW005 | KU986339 |
| *Bothrops barnetti* | EANAH425-12 | BIOUG-PW007 | KU986343 |
| *Bothrops brazili* | EANAH874-12 | ROM Herps 42604 | KU986320 |
| *Bothrops leucurus* | EANAH422-12 | BIOUG-PW004 | KU986310 |
| *Bothrops* sp. | EANAH442-12 | BIOUG-PW024 | KU986346 |
| *Corallus caninus* | EANAH453-12 | BIOUG-PW035 | KU986312 |
| *Crotalus durissus terrificus* | EANAH436-12 | BIOUG-PW018 | KU986344 |
| *Draco volans* | EANAH734-12 | ROM Herps 17474 | KU986311 |
| *Eryx jaculus* | EANAH850-12 | ROM Herps 23415 | KU986317 |
| *Melanosuchus niger* | EANAH808-12 | ROM Herps 23221 | KU986330 |
| *Phelsuma grandis* | EANAH433-12 | BIOUG-PW015 | KU986345 |
| *Phelsuma grandis* | EANAH449-12 | BIOUG-PW031 | KU986309 |
| *Python reticulatus* | EANAH733-12 | ROM Herps 17215 | KU986326 |
| *Rhadinophis oxycephalum* | EANAH448-12 | BIOUG-PW030 | KU986341 |
| *Tiliqua multifasciata* | EANAH875-12 | ROM Herps 43319 | KU986333 |
| *Trimeresurus albolabris* | EANAH421-12 | BIOUG-PW003 | KU986319 |
| *Trimeresurus albolabris* | EANAH861-12 | ROM Herps 35299 | KU986327 |
| *Trimeresurus trigonocephalus* | EANAH437-12 | BIOUG-PW019 | KU986306 |
| *Trimeresurus trigonocephalus* | EANAH438-12 | BIOUG-PW020 | KU986336 |
| *Tropidolaemus wagleri* | EANAH427-12 | BIOUG-PW009 | KU986318 |
| *Varanus prasinus horni* | EANAH851-12 | ROM Herps 24461 | KU986338 |
| *Vipera ammodytes* | EANAH456-12 | BIOUG-PW038 | KU986335 |
| *Vipera nikolskii* | EANAH876-12 | ROM Herps 43341 | KU986308 |
| *Vipera raddei* | EANAH446-12 | BIOUG-PW028 | KU986316 |
| *Vipera raddei* | EANAH447-12 | BIOUG-PW029 | KU986315 |
| *Vipera russelli russelli* | EANAH439-12 | BIOUG-PW021 | KU986321 |
| *Vipera russelli russelli* | EANAH440-12 | BIOUG-PW022 | KU986334 |
