# Supplementary material for: Assessing DNA Barcodes for Species Identification in North American Reptiles and Amphibians in Natural History Collections
Source: PLoS One. 2016 Apr 26;11(4):e0154363. doi: 10.1371/journal.pone.0154363 (PMC4846166; doi:10.1371/journal.pone.0154363)
Supplement: S2 Table — (DOCX) [file pone.0154363.s005.docx]

**S2 Table. Length of amplicon, DNA dilution factors, success rates, and sample sizes for all specimens (except formalin-fixed).**

| Primer set | Dilution factor | Amplicon (bp) | Success rate (sample size)  Amphibia Reptilia | | Overall success rate (sample size) |
| --- | --- | --- | --- | --- | --- |
| AmphF2_t1 + AmphR3_t1 | None | 658 | 31% (95) | 84% (520) | 76% (615) |
| Chmf(r)4 (Anura) | None | 658 | 44% (190) | N/A | 44% (190) |
| COI-C02/04 (Caudata) | None | 658 | 73% (95) | N/A | 73% (95) |
| MLepF1 + AmphR3_t1 | 10x | 407 | 70% (184) | 45% (120) | 60% (304) |
| AmphF2_t1 + MLepR2 | 100x | 307 | 44% (184) | 37% (120) | 41% (304) |
| MLepF2_t1 + microLepR2 | None | 295 | 16% (148) | 23% (104) | 19% (252) |
| AncientLepF2 + MLepR2 | None | 189 | 32% (148) | 26% (104) | 29% (252) |
