# Supplementary material for: Assessing DNA Barcodes for Species Identification in North American Reptiles and Amphibians in Natural History Collections
Source: PLoS One. 2016 Apr 26;11(4):e0154363. doi: 10.1371/journal.pone.0154363 (PMC4846166; doi:10.1371/journal.pone.0154363)
Supplement: S3 Table — (DOCX) [file pone.0154363.s006.docx]

**S3 Table. PCR regime details for primers used in this project.**

| Regime name | Regime | Primer sets |
| --- | --- | --- |
| COIFast | 94°C for 1min; 5x: 94°C for 40s, 45°C for 40s, 72°C for 1min; 35x: 94°C for 40s, 51°C for 40s, 72°C for 1min; 72°C for 5min | AmphF2_t1+AmphR3_t1; Chmf(r)4; COI-C02/04 |
| mini51 | 94°C for 1min; 40x: 94°C for 40s, 51°C for 40s, 72°C for 1min; 72°C for 5min | AmphF2_t1+MLepR2; MLepF1+AmphR3_t1 |
| micro60 | 94°C for 1min; 40x: 94°C for 40s, 60°C for 40s, 72°C for 1min; 72°C for 5min | MLepF2_t1+MicroLepR2; AncientLepF2+MLepR2 |
