# Supplementary material for: Assessing DNA Barcodes for Species Identification in North American Reptiles and Amphibians in Natural History Collections
Source: PLoS One. 2016 Apr 26;11(4):e0154363. doi: 10.1371/journal.pone.0154363 (PMC4846166; doi:10.1371/journal.pone.0154363)
Supplement: S4 Table — Ages are relative to year of sequencing (2012). (DOCX) [file pone.0154363.s007.docx]

**S4 Table. Specimens with collection date information included in linear regression analysis.** Ages are relative to year of sequencing (2012).

| Institution | Class | Sample size | Collection date range | Mean age (years) |
| --- | --- | --- | --- | --- |
| American Museum of Natural History | Amphibia | 7 | 2009-2010 | 2.29 |
|  | Reptilia | 23 | 2009-2010 | 2.09 |
| Field Museum of Natural History | Amphibia | 14 | 1993-2011 | 5.57 |
|  | Reptilia | 38 | 1989-2011 | 11.26 |
| Kansas University | Amphibia | 28 | 1987-2006 | 18.32 |
|  | Reptilia | 44 | 1989-2007 | 10.89 |
| Museum of Vertebrate Zoology | Amphibia | 54 | 1818-2005 | 32.33 |
|  | Reptilia | 39 | 1818-2004 | 29.16 |
| Royal Ontario Museum | Amphibia | 64 | 1900-1991 | 37.53 |
|  | Reptilia | 210 | 1970-1996 | 28.73 |
| San Diego Natural History Museum | Reptilia | 14 | 2011-2012 | 1.00 |
